# Herpes Simplex Virus Type 2 Infection-Induced Expression of CXCR3 Ligands Promotes CD4^+^ T Cell Migration and Is Regulated by the Viral Immediate-Early Protein ICP4

**DOI:** 10.3389/fimmu.2018.02932

**Published:** 2018-12-19

**Authors:** Mudan Zhang, Xu Deng, Xinmeng Guan, Lanlan Geng, Ming Fu, Binman Zhang, Rui Chen, Huimin Hu, Kai Hu, Di Zhang, Mei Li, Yalan Liu, Sitang Gong, Qinxue Hu

**Affiliations:** ^1^The Joint Center of Translational Precision Medicine, Guangzhou Institute of Pediatrics, Guangzhou Women and Children's Medical Center, Wuhan Institute of Virology, Chinese Academy of Science, Wuhan, China; ^2^State Key Laboratory of Virology, Wuhan Institute of Virology, Chinese Academy of Sciences, Wuhan, China; ^3^University of Chinese Academy of Sciences, Beijing, China; ^4^Department of Gastroenterology, Guangzhou Women and Children's Medical Center, Guangzhou Medical University, Guangzhou, China; ^5^Institute for Infection and Immunity, St George's University of London, London, United Kingdom

**Keywords:** HSV-2, CXCR3 ligands, CD4^+^ T cells, recruitment, ICP4

## Abstract

HSV-2 infection-induced CXCR3 ligands are important for the recruitment of virus-specific CD8^+^ T cells, but their impact on CD4^+^ T cell trafficking remains to be further determined. Given that recruitment of CD4^+^ T cells to infection areas may be one of the mechanisms that account for HSV-2 infection-mediated enhancement of HIV-1 sexual transmission, here we investigated the functionality of HSV-2 infection-induced CXCR3 ligands CXCL9, CXCL10, and CXCL11 *in vivo* and *in vitro*, and determined the viral components responsive for such induction and the underlying mechanisms. We first found that the expression of CXCR3 ligands CXCL9, CXCL10, and CXCL11 was increased in mice following vaginal challenge with HSV-2, while CXCL9 played a predominant role in the recruitment of CD4^+^ T cells to the vaginal foci of infected mice. HSV-2 infection also induced the production of CXCL9, CXCL10, and CXCL11 in human cervical epithelial cells. Of note, although HSV-2 induced the expression of all the three CXCR3 ligands, the induced CXCL9 appeared to play a predominant role in promoting CD4^+^ T cell migration, reflecting that the concentrations of CXCL10 and CXCL11 required for CD4^+^ T cell migration are higher than that of CXCL9. We further revealed that, ICP4, an immediate-early protein of HSV-2, is crucial in promoting CXCR3 ligand expression through the activation of p38 MAPK pathway. Mechanistically, ICP4 binds to corresponding promoters of CXCR3 ligands via interacting with the TATA binding protein (TBP), resulting in the transcriptional activation of the corresponding promoters. Taken together, our study highlights HSV-2 ICP4 as a vital viral protein in promoting CXCR3 ligand expression and CXCL9 as the key induced chemokine in mediating CD4^+^ T cell migration. Findings in this study have shed light on HSV-2 induced leukocyte recruitment which may be important for understanding HSV-2 infection-enhanced HIV-1 sexual transmission and the development of intervention strategies.

## Introduction

Herpes simplex virus type 2 (HSV-2), a large enveloped dsDNA virus, affects ~500 million people worldwide and acquires an annual rate of close to 25 million (1), resulting in up to 40% human adults living with HSV-2 latency (2, 3). HSV-2 infections are known to be restricted to mucosal and keratinized epithelia and neuronal ganglia, and cause genital herpes (4) with sexual transmission being the main route (5). Human immunodeficiency virus type 1 (HIV-1) causes destruction of the immune system, leading to acquired immune deficiency syndrome (AIDS) (6). In 2016, there were 1.8 million new HIV-1 infections globally, adding up to a total of 36.7 million people living with HIV-1 (7). The majority of HIV-1 infections are acquired by genital mucosal exposure, with sexual transmission as the leading mode of HIV-1 infection worldwide (8).

Due to the high positive-incidence of HSV-2 and common routes of transmission with HIV-1, mucosal HIV-1/HSV-2 co-infections attract more and more attention. Epidemiological studies show that HSV-2 infection results in an ~3-fold increased risk of HIV-1 acquisition (9, 10), but the underlying mechanisms remain to be determined. One of the mechanisms that HSV-2 infection increases the probability of HIV-1 acquisition is the generation of lesions at HSV-2-infection sites, which provides a chance for HIV-1 to contact the target cells in the epidermis and dermis (11). Moreover, the number of CD4^+^ T cells at the infection sites is increased following HSV-2 infection, which may further facilitate HIV-1 to infect these target cells (12–14). However, it is still not fully elucidated concerning the mechanism of CD4^+^ T cell migration induced by HSV-2.

Chemokine CXCL9 is a member of the CXC family and plays an important role in the chemotaxis of CXCR3^+^ immune cells. CXCR3 is a chemokine receptor that is rapidly induced on activated naive cells and sustains highly expression on Th1-type CD4^+^ T cells and effector CD8^+^ T cells (15). CXCR3 could be activated by three interferon-inducible ligands CXCL9 (MIG), CXCL10 (IP-10), and CXCL11 (I-TAC). Although CXCR3 could also be activated by CXCL4 and CXCL4L1, these two chemokines are released by platelets and have been implicated in atherogenesis and acute coronary syndrome (16). It is known that the upregulation of CXCR3 ligands is positively associated with a variety of tumors, inflammatory diseases, and infectious diseases such as AIDS (17). Although the expression of CXCL9 and CXCL10 has been shown to be increased in the cervical tissues of mice infected by HSV-2, the study on mice focused on the roles of recruited CD8^+^ T cells in control of HSV-2 infection (18–20). Our previous study demonstrated that CXCL9 levels in cervical mucus from HSV-2–positive women were significantly increased and that CXCL9 induced by HSV-2 infection in cervical epithelial cells can enhance the migration of CD4^+^ T cells (14). Although HSV-2-induced expression of CXCL10 and CXCL11 was previously reported (19, 21), the significance of HSV-2-induced CXCR3 ligands *in vivo* and the molecular mechanisms underlying the HSV-2-induced expression of CXCR3 ligands, in particular CXCL10 and CXCL11, have yet to be addressed. Furthermore, HSV-2 component(s) responsible for the induction and the underlying mechanism remain to be fully investigated.

In the current study, we found that expression of mouse CXCR3 ligands was increased following vaginal challenge with HSV-2 in mice. In addition, HSV-2-induced CXCL9 played a crucial role in promoting CD4^+^ T cell migration to the vaginal foci of infected mice. In human cervical epithelial cells, HSV-2 infection induced the production of CXCL10 and CXCL11 in addition to CXCL9. Although CXCL10 and CXCL11 were induced following HSV-2 infection, the migration of CD4^+^ T cells was mainly dependent on HSV-2 infection-induced CXCL9, reflecting that the concentrations of CXCL10 and CXCL11 required for CD4^+^ T cell migration are higher than that of CXCL9. Moreover, HSV-2 immediate-early protein ICP4 (also known as RS1) appeared to be the vital viral component to induce the production of CXCR3 ligands. We further explored the molecular mechanisms underlying ICP4–induced CXCR3 ligand expression, revealing that ICP4 binds to corresponding promoters of CXCR3 ligands to activate their transcription by interaction with TBP. Our study together has shed light on the molecular mechanisms underlying HSV-2-induced CD4^+^ T cell accumulation in mucosal infection sites, which may be crucial for understanding HSV-2 infection-enhanced HIV-1 sexual transmission and the development of intervention strategies.

## Materials and Methods

### Viruses, Cell Lines, Antibodies, and Inhibitors

HSV-2 (G strain) was obtained from LGC standards and propagated in African green monkey kidney cells (Vero). Virus stocks were aliquoted and stored at −80°C before used for infection. Ultraviolet (UV)-inactivated HSV-2 was obtained by exposure to ultraviolet irradiation for 15 min. HSV-2 titration was determined by plaque assay on confluent Vero monolayers (53). ME180, PM1, and Vero cells were obtained from American Tissue Culture Collection. Human cervical epithelial cell line ME180 and Vero cells were cultured in Dulbecco's modified Eagle medium (DMEM) (Life Technologies, 11965, Australia) supplemented with 10% FBS, 100 units/mL penicillin and 100 units/mL streptomycin at 37°C in a 5% CO2 incubator. Human T cell line PM1 cells were cultured in RPMI-1640 medium (HyClone, SH30809.01B, USA) supplemented with 10% FBS, 100 units/mL penicillin and 100 units/mL streptomycin at 37°C in a 5% CO2 incubator. Abs against p38, phospho-p38, and β-actin, respectively, were purchased from Santa Cruz Biotechnology (sc-7149, sc-101759 and sc-81178, USA). Ab against phospho-C/EBP-β was purchased from Cell Signaling Technology (3084S, USA). Inhibitors specifically against ERK (PD98059), JNK (SP600125), and p38 (SB203580), respectively, were purchased from Merck Millipore (19-143, 420119, and 559389, USA). Abs against HA and Flag tag were purchased from Sigma-Aldrich (H6908 and F1804, USA). Ab against Proliferating Cell Nuclear Antigen (PCNA) and TATA binding protein (TBP) were from Proteintech (10205-2-AP and 22006-1-AP, Wuhan, China). Rabbit normal IgG and Cy3-conjugated goat anti-mouse IgG were purchased from BOSTER (BA1031 and BA1045, Wuhan, China). Abs against mouse CD4, CXCL9, CXCL10, and CXCL11 were purchased from R&D Systems (MAB554, AF-492-NA, AF-466-NA, and AF-572, USA). Abs against ICP4, ICP27, gB, and HSV-2 were from Abcam (ab96431, ab53480, ab6506, and ab21112, England). Ab against gD was from Santa Cruz Biotechnology (sc-69802, USA).

### Plasmid Construction

HSV-2 genome was extracted from the cells infected with HSV-2 for 48 h using QIAamp DNA Blood Mini Kit (Qiagen, 51104, Germany). The expression plasmids of US1, RS1, US12, UL54, and RL2, and the reporter of CXCL9 were described previously (14, 22). The open reading frames (ORFs) were amplified by PCR with the primers shown in Table S1. The reporters of CXCL10 and CXCL11 were amplified with forward primers (CXCL10 Luc-F and CXCL11 Luc-F) and reverse primers (CXCL10 Luc-R and CXCL11 Luc-R), respectively. The sequences of primers were showed in Table S1. An N-terminal HA or Flag tag was introduced into ICP4 by the forward primer. N-terminal Flag tag was introduced into UL20, UL46, UL47, UL48, UL56, UL49A, US4, US7, or RL1 by the forward primer. The promoter reporters were cloned into pGL3-basic. Unless otherwise described, other PCR products were cloned into pcDNA3.1(+) (Invitrogen) and the constructed expression plasmids were named UL20, RS1-HA (ICP4-HA), RS1-Flag (ICP4-Flag), UL46, UL47, UL48, UL56, UL49A, US4, US7, RL1, UL20-Flag, UL46-Flag, UL47-Flag, UL48-Flag, UL56-Flag, UL49A-Flag, US4-Flag, US7-Flag, and RL1-Flag, respectively. The constructs were verified by DNA sequencing (Sunny Biotechnology, Shanghai, China).

### HSV-2 Challenge and Sampling

Animal experiments were approved by the Institutional Animal Care and Use Committee and performed in accordance with the guidelines of the Hubei Laboratory Animal Science Association. In brief, female BALB/c mice (6–8 wk old) were purchased from Beijing HFK Biotechnology (Beijing, China) and maintained in specific pathogen–free conditions with food and water supplied. Seven days prior to challenge, each mouse was injected with 2 mg progesterone in intraperitoneal, subcutaneous, and intramuscular sites to ensure that each mouse rapidly entered the estrous cycle (23). After the estrous cycle, the mouse vaginal mucosal epithelia became thinner and were more susceptible to HSV-2. One day prior to challenge, the neutralizing Abs against CXCL9 (2 μg, R&D Systems, MAB554, USA), CXCL10 (2 μg, R&D Systems, MAB554, USA), and CXCL11 (2 μg, R&D Systems, MAB554, USA) were delivered to the vagina of mice, respectively, or in combination. Mice were anesthetized with pentobarbital sodium and challenged intravaginally with 10 μL/mouse HSV-2 at a concentration of 6 × 10^7^ PFU/mL. Mice challenged with medium alone were set as background controls. The signs of mouse vagina were observed at days 3, 5, and 7 post HSV-2 challenge. Vaginal ulcers arose at day 7 in infected mice but not in the control group. Seven days after challenge, vaginal lavage fluids were collected using a vaginal Transferpettor by washing the vagina three times with sterile PBS plus protease inhibitors (Roche, 11697498001, Germany) in a total volume of 100 μL/mouse. Collected samples were centrifuged (15,000 × g, 10 min at 4°C), and supernatants were aliquoted and stored at −80°C until use. Thereafter, mice were sacrificed by neck dislocation. The cervical-vaginal tissues (Y type, two fallopian tube in the upper and vagina in the lower) were excised according to the characteristics of mouse physiological structure and collected under sterile conditions. The tissues were fixed in 4% formaldehyde followed by immunohistochemistry analysis. The collection of vaginal lavages or tissues was performed by the same people.

### CBA for Human CXCL9, CXCL10 and CXCL11, and Mouse CXCL9 and CXCL10

ME180 cells in 6-well plates were transfected with empty vector or plasmid expressing ICP4 for 24 h. In some cases, ME180 cells were infected or mock-infected with HSV-2 for 24 h. Cell supernatants were collected and centrifuged to remove cell debris. Cytometric Bead Assay (CBA) was carried out to quantify secreted human CXCL9, CXCL10, and CXCL11 using the BD Cytometric Bead Array Human Soluble Protein Flexset Kit according to the manufacturer's instructions. Briefly, 50 μL diluted standards or undiluted samples were added into labeled tubes followed by the addition of 50 μL mixed beads. At 1 h post-incubation, 50 μL PE conjugated detection antibody was added into all tubes followed by incubation for 2 h at room temperature. All tubes were then washed with 1 mL washing buffer and centrifuged at 1,200 rpm for 5 min. The supernatants were removed and the beads were resuspended with 300 μL washing buffer. The concentration of mouse CXCL9 and CXCL10 in the vaginal lavage fluids was detected using the LEGENDplex™ Cytometric Bead Array mouse proinflammation Chemokine Mix and Match Subpanel according to the manufacturer's instructions. Briefly, 25 μL assay buffer was added into all tubes, followed by the addition of 25 μL diluted standard or 25 μL undiluted sample to each labeled tube. Thereafter, 25 μL mixed beads and 25 μL detection antibodies were added into all tubes followed by incubation for 2 h at room temperature with shaking. All the tubes were then incubated for 30 min at room temperature after the addition of 25 μL SA-PE solution. Beads were spun down (1,100 rpm, 5 min at room temperature) and washed with 1 × washing buffer. The beads were resuspended with 200 μL of 1 × washing buffer. All the samples were read on the BD LSRFortessa™ Flow Cytometer.

### ELISA for Mouse CXCL11

The concentration of CXCL11 in the vaginal lavage fluids of mice was detected using Mouse CXCL11 ELISA Kit (BOSTER, EK0738, China). The standard of CXCL11 was provided in the Kit. Fifty microliter of undiluted fluids were tested for mouse CXCL11 detection according to the manufacturer's instructions.

### Immunohistochemistry

Immunohistochemistry analysis of mouse cervical-vaginal tissues was conducted as described previously (24, 25). Briefly, the specimens obtained from challenged mice were fixed in 4% formaldehyde for 24 h at room temperature, embedded in paraffin, and cut into 3-mm sections. For detection of CD4^+^ T cells in cervical-vaginal samples, slides were first dewaxed in xylene and rehydrated in a descendant ethanol scale. Ag retrieval was subsequently performed using Antigen Retrieval Reagent Basic Kit (R&D Systems, CTS013, USA) for 30 min in a water bath according to the manufacturer's instructions, and endogenous peroxidase was blocked by 3% H_2_O_2_ for 10 min at room temperature. Immunohistochemistry staining was performed using Cell & Tissue Staining Kit (R&D Systems, CTS017, USA) according to the manufacturer's instructions. CD4^+^ T cells were detected by rabbit anti-mouse CD4 Ab. HSV-2 infection was detected by goat anti-HSV-2 polyclonal Ab. The colorimetric reaction was developed by adding 3, 3′-diaminobenzidine (DAB) at room temperature. For immunofluorescence detection of ICP4 and CXCR3 ligands or HSV-2 in cervical-vaginal samples, slides were first treated as the above instruction. ICP4 was detected with rabbit anti HSV-2 ICP4 Ab. Mouse CXCL9, CXCL10, and CXCL11 were detected by goat anti-mouse CXCL9, CXCL10, and CXCL11 Abs, respectively. HSV-2 was detected by goat anti-HSV-2 polyclonal Ab. FITC-conjugated goat anti-mouse, Cy3 conjugated donkey anti-goat and anti-rabbit (Beyotime, A0568, A0502, and A0516, China) secondary Abs were used in subsequent detection. The images were acquired using the Hungary 3DHISTECH apparatus (Pannoramic MIDI).

### Dual Luciferase Report (DLR) Assay

ME180 cells were seeded in 24-well plates overnight and co-transfected with empty vector or plasmid encoding ICP4, Renilla luciferase plasmid phRL-TK and reporter plasmid CXCL9-Luc, CXCL10-Luc or CXCL11-Luc. Transfections were carried out using X-tremeGENE™ HP DNA Transfection Reagent (Roche, 6366236001, Germany) according to the manufacturer's instructions. At 24 h post-transfection, cells were harvested and lysed. The lysates were used for measuring firefly and Renilla luciferase activities using the Dual-Luciferase Reporter Assay System (Promega, E1980, USA) according to the manufacturer's instructions. For some experiments, ME180 cells were co-transfected with reporter plasmid CXCL9-Luc, CXCL10-Luc or CXCL11-Luc, and phRL-TK, followed by infection with HSV-2 or ultraviolet-inactivated HSV-2 at an MOI of 1. At 24 h post-infection, the enzymatic activities of Firefly and Renilla luciferase were measured. Values for the samples were normalized using Renilla luciferase values and expressed as fold increase of the value induced in cells transfected with empty vector or mock-infected with DMEM.

### RNA Isolation and Quantitative PCR

Cells were collected and total RNA was extracted using RNA isolation kit (MN, 740955, Germany) according to the manufacturer's instructions. The cDNA was synthesized by Moloney murine leukemia virus transcriptase (Promega, M170B, USA). The newly synthesized cDNA was used as the template for amplifying the genes of CXCR3 ligands and GAPDH. The primer pairs for CXCL9, CXCL10, and CXCL11 were named CXCL9-F/CXCL9-R, CXCL10-F/CXCL10-R, and CXCL11-F/CXCL11-R (Table S1). GAPDH was used as an internal control and amplified with primers GAPDH-F and GAPDH-R (Table S1). Relative real-time quantitative PCR was performed on an ABI StepOne apparatus using a SYBR Green Real-Time PCR Master Mix (Toyobo, QPK-201, Japan) according to the following conditions: 95°C for 1 min, followed by 40 cycles of 95°C for 15 s, 60°C for 15 s, and 72°C for 45 s. The expression difference was calculated on the basis of 2^−ΔΔ*Ct*^ values.

### Western Blot

Western blot analysis was performed as described previously (22). Briefly, cytoplasmic and nuclear proteins were isolated using the Nucleus and Cytoplasm Protein Extraction Kit (Beyotime, P0028, China). In some cases, cells were lysed with lysis buffer (Life technologies, 87788, USA). Cell extracts were subjected to 10 or 15% SDS-PAGE and transferred onto PVDF membranes (Millipore 0.45 μm or 0.22 μm) followed by blocking with 5% non-fat milk in Tris-buffered saline-Tween (TBST, 50mM Tris-HCl pH 7.5, 200mM NaCl, 0.1% (v/v) Tween-20) at room temperature for 2 h. The membrane was clipped according to the molecular weight of the protein, and then probed with an appropriate primary antibody at room temperature for 2 h. After three washes with TBST, the membrane was incubated with horseradish peroxidase (HRP)-conjugated goat anti-rabbit IgG (BOSTER, BA1054, China), goat anti-mouse IgG (BOSTER, BA1051, China) or donkey anti-goat IgG (Beyotime, A0181, China) at room temperature for 1 h. Protein bands were visualized by exposure to FluorChem HD2 Imaging System (Alpha Innotech) after the addition of chemiluminescent substrate (Beyotime, P0018, China). Protein molecular weight markers were purchased from Thermo Fisher (26616, USA) and YEASEN (20352, China).

### Isolation and Culture of PBMCs and CD4^+^ T Cells

All protocols involving human subjects were reviewed and approved by the local Research Ethics Committee of Wuhan Institute of Virology, Chinese Academy of Sciences. Informed written consents from the human subjects were obtained in this study. Both sexes were used and the donors were free of HSV-1 and HSV-2. PBMCs were isolated from healthy donors by using a Ficoll-Hypaque density gradient. CD4^+^ T cells were separated from PBMCs using CD4^+^ Cell Negative Isolation Kit according to the manufacturer's protocol (Miltenyi Biotec, 130-096-533, Germany). PBMCs and CD4^+^ T cells were activated by stimulating with 1 μg/mL PHA (Sigma-Aldrich, L4144, USA) and 20 U/mL IL-2 (PeproTech, 200-02, USA). PBMCs and CD4^+^ T cells cultured in complete RPMI 1640 containing 20 U/mL IL-2 were used as controls for flow cytometry. PBMCs and CD4^+^ T cells were harvested at day 7 and used in subsequent assays.

### Chemotaxis Assay

Chemotaxis assay was performed using 24-well Transwell plates (Costar, 3415, USA). One milliliter supernatants from ME180 cells which were mock-infected or infected with HSV-2, or mock-transfected or transfected with ICP4 expressing plasmid were added to the lower chamber. To examine the roles of CXCR3 and CXCR3 ligands in mediating cell migration, supernatants or cells were incubated with anti-CXCL9 (10 μg/mL, R&D Systems, MAB392, USA), -CXCL10 (2 μg/mL, R&D Systems, MAB266, USA), -CXCL11 (2 μg/mL, R&D Systems, MAB672, USA) or -CXCR3 (1 μg/mL, R&D Systems, MAB160, USA) neutralizing Abs, respectively, for 1 h, according to the manufacturer's instructions. Activated PBMCs and CD4^+^ T cells (5 × 10^5^) in 100 μL RPMI-1640 medium were added to the upper chamber. The chambers were incubated for 2 h at 37°C in a 5% CO2 incubator. Cell migrated to the lower chambers were collected and counted using an automatic cell counter (Bio-Rad).

### Flow Cytometry

PBMCs and CD4^+^ T cells were collected and resuspended with 3% FBS on ice for 10 min. Hundred microliter cell suspension (1 × 10^6^) was prepared for one test. Two microliter BV421 conjugated mouse anti-human CD25 (BD biosciences, 562443, USA), BB515 conjugated mouse anti-human CD4 (BD biosciences, 564419, USA) and APC conjugated mouse anti-human CD69 (BD biosciences, 560967, USA) Abs or PE conjugated mouse anti-human CXCR3 Ab (BD biosciences, 560928, USA) were added into the corresponding samples, followed by incubation on ice for 15 min. Background staining was assessed by isotype-matched control Abs, including BV421 conjugated mouse IgG1 (BD biosciences, 562438, USA), BB515 conjugated mouse IgG1 (BD biosciences, 564416, USA), APC conjugated mouse IgG1 (BD biosciences, 555751, USA), and PE conjugated mouse IgG1 (BD biosciences, 555749, USA). Cells were washed with 1 × PBS for three times. Three hundred microliter cell suspension was filtrated through a 200-mesh membrane and performed on BD LSRFortessa™ Flow Cytometer. Data were analyzed using BD FACSDiva software (BD Biosciences).

### Immunofluorescence Assay

ME180 cells were seeded in 35-mm dishes with glass bottom and transfected with HA-tagged plasmid expressing ICP4. At 24 h post-transfection, cells were fixed with 4% formaldehyde and permeabilized with 0.2% Triton X-100. After three washes with 1 × PBS, cells were blocked in PBS containing 5% BSA at 4°C overnight. Thereafter, cells were incubated with mouse anti-HA Ab at a dilution of 1:100 at 37°C for 1 h. Following three washes with 1 × PBS, cells were then incubated with Cy3-conjugated goat anti-mouse IgG (Beyotime, A0521, China) at a dilution of 1:50 for 1 h at 37°C. Cells were subsequently washed and incubated with DAPI for 10 min at 37°C. After washes, cells were incubated with anti-fluorescence quenching reagent (Beyotime, P0126, China) and observed under a fluorescence microscope (Olympus IX51).

### Chromatin Immunoprecipitation (ChIP)

ME180 cells in 6-well plates were transfected with HA-tagged plasmid expressing ICP4 or empty vector. At 24 h post-transfection, ChIP assay was performed as described previously (22) according to the manufacturer's instructions (Millipore, 17-409, Germany). The purified DNA was used as a template for PCR detection of the promoter sequences of CXCR3 ligands with primer pairs CXCL9 pro-F/CXCL9 pro-R, CXCL10 pro-F/CXCL10 pro-R, and CXCL11 pro-F/CXCL11 pro-R, respectively (Table S1).

### Co-immunoprecipitation (Co-IP) Assay

ME180 cells in 6-well plates were transfected with HA-tagged ICP4 expression plasmid or empty vector. At 24 h post-transfection, cells were harvested and lysed on ice for 10 min in 200 μL of lysis buffer (50 Mm Tris (PH 8.0), 150 mM NaCl, 1% NP40) containing protease inhibitor cocktail (Roche, 11697498001, Germany). To eliminate nonspecific binding of other proteins, the samples were pretreated with dynabeads Protein G (Invitrogen, 10003D, USA) for 2 h at room temperature followed by separation prior to Co-IP assay. Meanwhile, 2 μg rabbit anti-HA Ab or control rabbit Ab was diluted in 200 μL PBS with 1% Tween-20 (PBST) and added to fresh dynabeads protein G. After incubation with rotation for overnight at 4°C, dynabeads-Ab complexes were washed once with 200 μL PBST before mixed with the pretreated samples, followed by overnight incubation with rotation at 4°C to allow the formation of dynabeads-Ab-Ag complexes. The complexes were washed three times with PBST and target antigens were eluted by boiling and subjected to western blot analysis.

### Statistical Analysis

All experiments were repeated at least three times and the data are presented as mean ± S.D. with each condition performed in triplicate or in duplicate unless otherwise specified. Data analyses were performed with GraphPad Prism 5 software (GraphPad). Comparison between two groups was analyzed by two tailed unpaired Student's *t*-test, whereas comparisons among more than two groups were analyzed by one-way ANOVA with the Turkey's test. *P* < 0.05 was considered statistically significant.

## Results

### Contribution of HSV-2 Infection-induced CXCR3 Ligands to CD4^+^ T Cell Infiltration Into Mouse Vagina

**Figure 1:**
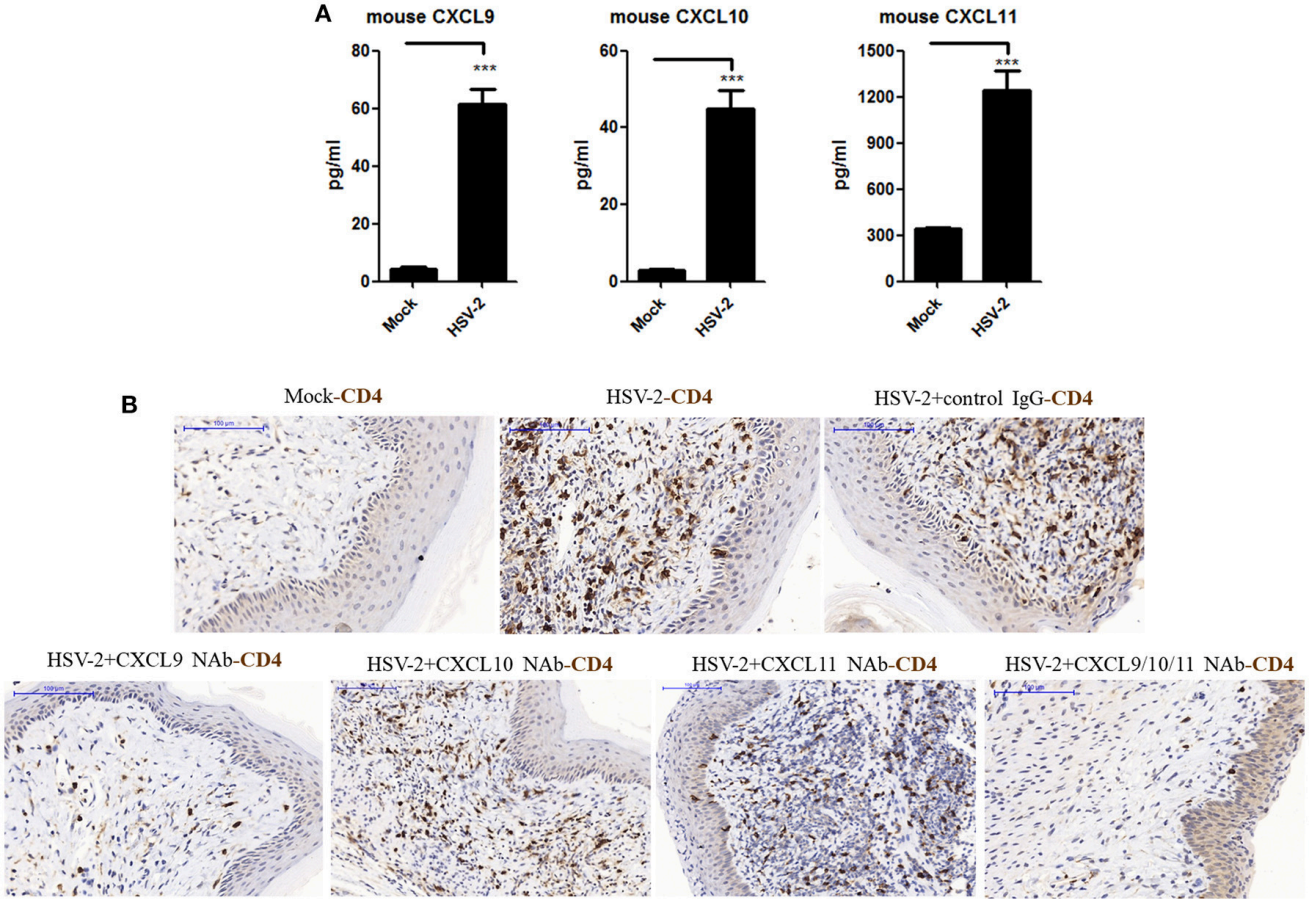
Contribution of HSV-2 infection-induced CXCR3 ligands to CD4^+^ T cell infiltration into mouse vagina. Seven days prior to HSV-2 challenge, BALB/c mice were injected with progesterone in multiple sites. One day prior to HSV-2 challenge, CXCL9, CXCL10, and CXCL11 neutralizing antibodies were delivered to the vagina of mice, alone or in combination, while isotype matched control IgG was used as the control. Mice were then anesthetized with pentobarbital sodium and challenged intravaginally with 10 μL/ mouse HSV-2 at a concentration of 6 × 10^7^ PFU/ml or mock- challenged. Vaginal lavage fluids and cervical-vaginal tissues were collected at day 7 after challenge. **(A)** HSV-2 infection induces the production of mouse CXCR3 ligands. The protein levels of CXCL9 and CXCL10 ligands in vaginal lavage fluids were measured by CBA, and the protein level of CXCL11 was detected by ELISA. **(B)** CXCL9 mediates the migration of CD4^+^ T cells to the vaginal foci of infected mice. CD4^+^ T cells in infection foci were detected using anti-CD4 Ab by IHC. The scale bar indicates 100 μm. Data shown are mean ± S.D. (*n* = 5 mice/group) of three independent experiments **(A)**. ***p < 0.001. One representative out of three independent experiments is shown **(B)**.

The expression of CXCL9 and CXCL10 has been shown to be increased in the cervical tissues of mice infected by HSV-2 in previous studies (20), which mainly focused on the recruitment of activated CD8^+^ T cells and its contribution to the control of HSV-2 infection. However, the impact of HSV-2 infection on CD4^+^ T cell migration in mice remains to be further addressed. To assess this, mice were challenged with HSV-2 vaginally, and vaginal lavage fluids and cervical-vaginal tissues of the mice were collected for subsequent assessment. We confirmed a productive infection of HSV-2 in mouse vagina by immunohistochemistry and immunofluorescence-histochemistry assays (Supplementary Material Figures 1A,B), while Cytometric Bead Array (CBA) showed that the production of mouse chemokines CXCL9 and CXCL10 was significantly increased (Figure 1A). ELISA also indicated the enhancement of CXCL11 in mice challenged with HSV-2 (Figure 1A). Meanwhile, immunohistochemistry (IHC) assays showed that the number of CD4^+^ T cells was significantly increased in the vaginal foci of infected mice (Figure 1B). To identify which chemokine plays a vital role in CD4^+^ T cell recruitment, mice were vaginally treated with the neutralizing Ab against CXCL9, CXCL10, or/and CXCL11 before HSV-2 challenge. The number of CD4^+^ T cells was dramatically decreased after the administration of CXCL9 neutralizing antibody to the vagina of mice (Figure 1B). Although the migration of CD4^+^ T cells was almost completely abolished after administration of a combination of neutralizing Abs against CXCL9, CXCL10, and CXCL11 into the vaginal tissue, CD4^+^ T cells were still significantly recruited to the infection foci after the administration of CXCL10 or CXCL11 neutralizing antibody (Figure 1B). These data together indicate that HSV-2 vaginal infection of mice increases the expression of CXCR3 ligands CXCL9, CXCL10 and CXCL11, and the migration of CD4^+^ T cells to the vaginal foci is mainly mediated by CXCL9.

### HSV-2 Infection Induces the Production of CXCR3 Ligands in Human Cervical Epithelial Cells

**Figure 2:**
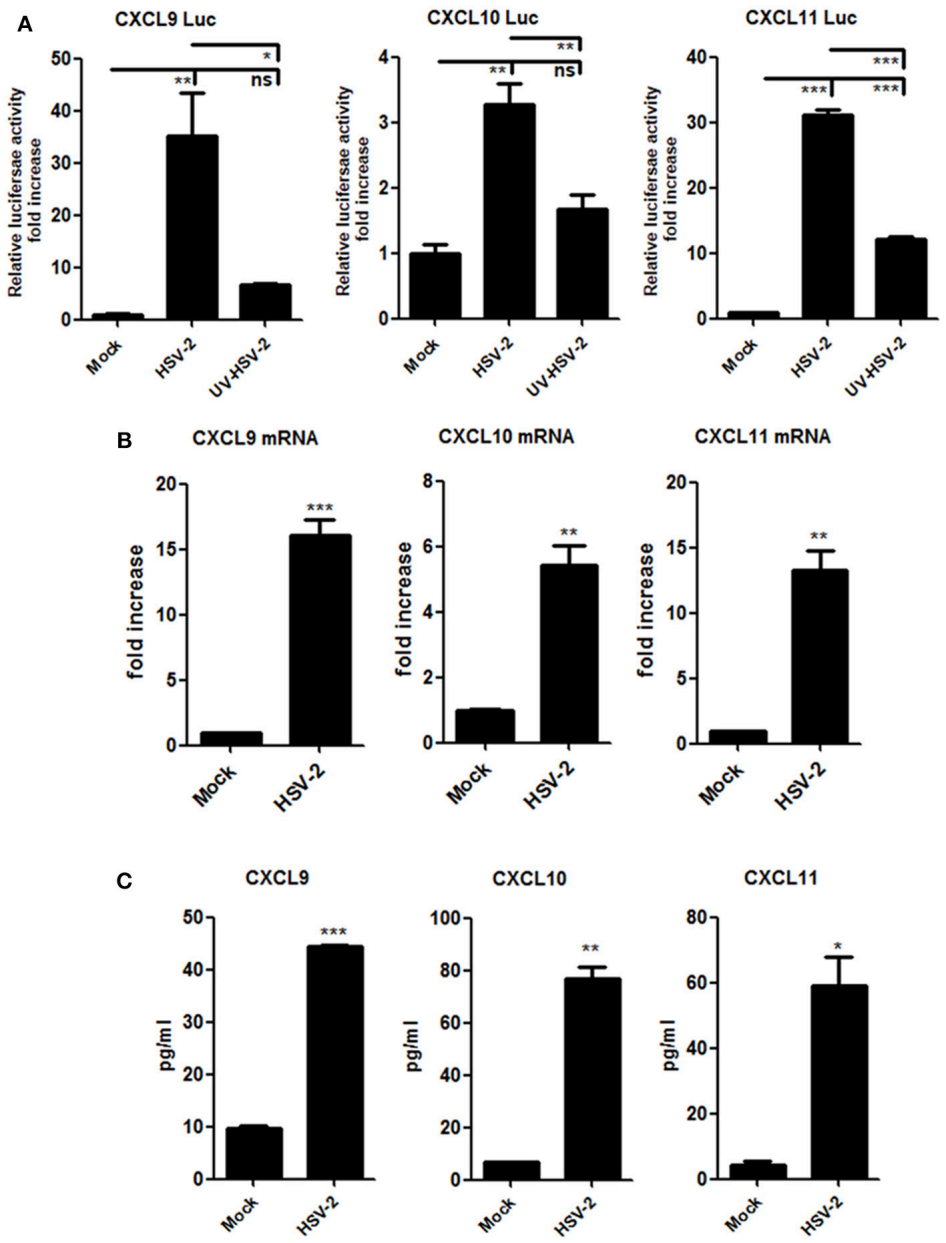
HSV-2 infection induces the production of CXCR3 ligands in human cervical epithelial cells. **(A)** HSV-2 infection activates the promoters of human CXCR3 ligands. ME180 cells in 24-well plates were co-transfected with 150 ng CXCL9-Luc, CXCL10-Luc or CXCL11-Luc, and 15 ng internal control plasmid phRL-TK. At 4 h post-transfection, cells were infected with HSV-2 or ultraviolet-inactivated HSV-2 (UV-HSV-2) at an MOI of 1 for 24 h. DLR assay was performed. Values for the samples were normalized using Renilla luciferase values and expressed as fold increase of the value induced in mock-infected samples. **(B)** HSV-2 infection induces the mRNA production of CXCR3 ligands. ME180 cells in 6-well plates were infected with HSV-2 at an MOI of 1 for 24 h. Cells were harvested and total RNA was extracted. The expression of CXCR3 ligands and GAPDH was evaluated by relative real-time quantitative PCR. The Ct values of GAPDH among all groups were equable and not overloaded. mRNA copies of CXCR3 ligands were normalized using GAPDH and expressed as fold increase of the value for the mock-infected control. **(C)** HSV-2 infection induces the production of CXCR3 ligands. As depicted in **(B)**, cell supernatants were collected, and the protein level of CXCR3 ligands was measured by CBA. Data shown are mean ± S.D. of three independent experiments (A, B, and C). *p < 0.05, **p < 0.01, ***p < 0.001.

Although HSV-2-induced expression of CXCL10 and CXCL11 was previously reported (19, 21), it remains to be addressed as to how HSV-2 induces the expression CXCR3 ligands in human mucosal epithelial cells. Epithelial cells are the primary HSV-2 target cells during sexual transmission. Having demonstrating the correlation of CXCR3 ligands with CD4^+^ T cell migration in mice, we next addressed the underlying mechanism in cellular models. Our previous study showed that HSV-2 infection of human epithelial cells induces CXCL9 expression (14). To investigate the association between HSV-2 infection and the induction of CXCR3 ligands, human cervical epithelial cell line ME180 was used for assessing CXCR3 ligand expression at promoter, mRNA and protein levels. Our results indicated that HSV-2 infection significantly activated not only the promoter of CXCL9 but also the promoters of CXCL10 and CXCL11 (Figure 2A). We next assessed whether HSV-2 productive infection is necessary for the transcriptional activation of CXCR3 ligands. The results showed that UV-inactivated HSV-2 did not significantly induce the transcriptional activation of CXCL9 and CXCL10. Although CXCL11 appeared to be induced by UV-inactivated HSV-2, the level of induction was low (Figure 2A). In agreement, several HSV-2 proteins were undetectable following UV inactivation (Supplementary Material Figure 2). These results together indicated that HSV-2 productive infection is essential for the induced production of CXCR3 ligands. To further confirm the effect of HSV-2 on the induction of CXCL10 and CXCL11, we next investigated the mRNA and protein levels of CXCL10 and CXCL11 following HSV-2 infection. Relative real-time PCR assay and CBA showed that HSV-2 infection significantly promoted the production of CXCL9, CXCL10, and CXCL11 at both mRNA (Figure 2B) and protein levels (Figure 2C).

### HSV-2 Infection-induced CXCL9 Plays a Predominant Role in Mediating CD4^+^ T Cell Migration

**Figure 3:**
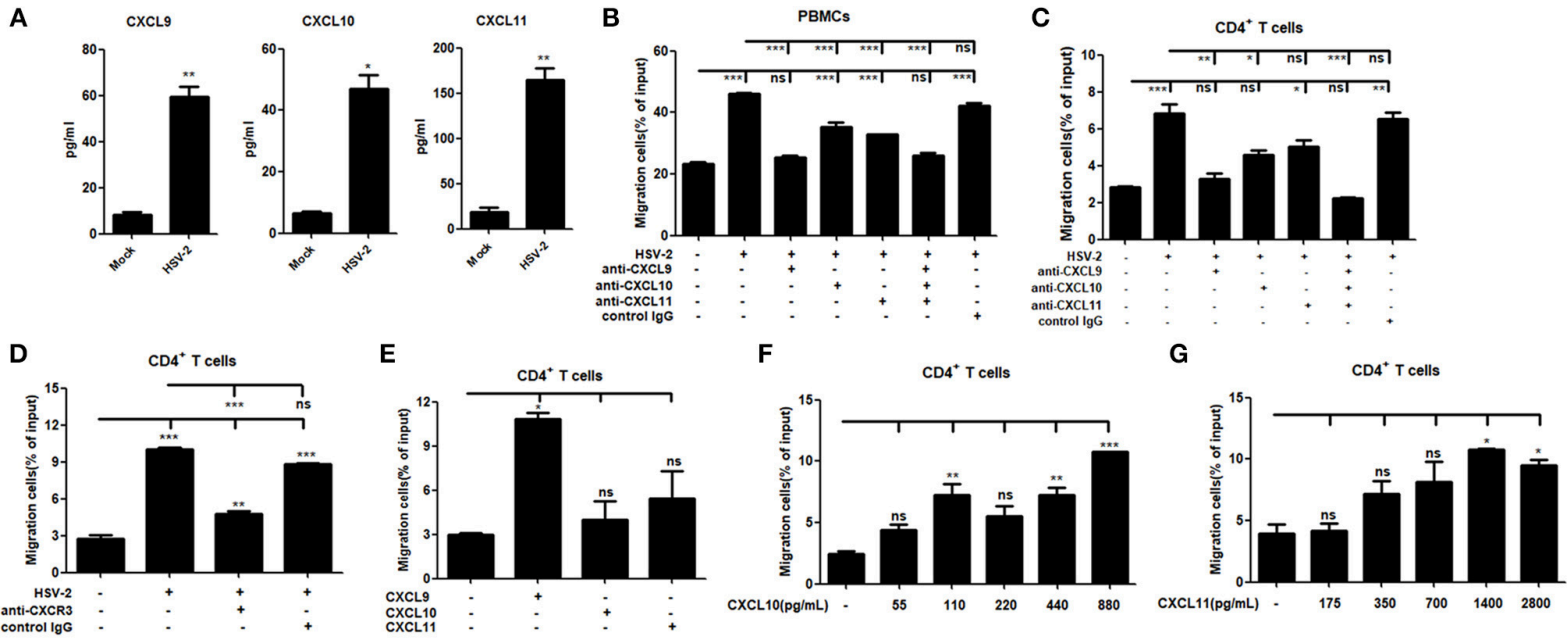
HSV-2 infection-induced CXCL9 plays a predominant role in mediating CD4^+^ T cell migration. **(A)** The concentrations of CXCR3 ligands in the supernatants of ME180 cells infected with HSV-2 or mock-infected with DMEM were detected by CBA. **(B,C)** CXCL9 induced by HSV-2 recruits the migration of PBMCs **(B)** and CD4^+^ T cells **(C)**. ME180 cells in 6-well plates were infected with HSV-2 at an MOI of 1 for 24 h. Cell supernatants were collected and added to the lower chamber of transwell plates in the absence or presence of anti-CXCL9, –CXCL10, or/and –CXCL11 neutralizing Ab or control Ab for 1 h. **(D)** Neutralization of CXCR3 reduces the migration of CD4^+^ T cells induced by HSV-2 infection. ME180 cells in 6-well plates were infected with HSV-2 at an MOI of 1 for 24 h. Cell supernatants were collected and added to the lower chamber of transwell plates. The activated CD4^+^ T cells were incubated with RPMI 1,640 medium containing anti-CXCR3 neutralizing Ab for 1 h and placed in the upper chamber. **(E)** Recombinant CXCL9 significantly induces the migration of CD4^+^ T cells. DMEM containing recombinant CXCL9, CXCL10, or CXCL11 (48 pg/mL, 55 pg/mL and 175 pg/mL, respectively; the lowest concentration induced by HSV-2 infection) was added to the lower chamber of transwell plates. **(F,G)** Recombinant CXCL10 or CXCL11 mediates the migration of CD4^+^ T cells in a dose-dependent manner. DMEM containing recombinant CXCL10 or CXCL11 was added to the lower chamber of transwell plates. CXCL10 or CXCL11 was started from 55 pg/mL and 175 pg/mL, respectively, at a concentration gradient of two times. The activated CD4^+^ T cells were placed in the upper chamber. After 2 h incubation, cells migrated to lower chambers were collected and counted using an automatic cell counter. Cells migration was expressed as percentage of input. Input cells in the upper chamber were 5 × 10^5^. Data shown are mean ± S.D. of three independent experiments **(A–G)**. ns, not significant, *p < 0.05, **p < 0.01, ***p < 0.001.

It is known that CXCR3 is highly expressed on CD4^+^ T cells and CXCL9, CXCL10 or CXCL11 could activate CXCR3^+^ T cells (15). We previously demonstrated the functionality of HSV-2-induced CXCL9 in chemotacting CD4^+^ T cells (14). To assess the functionality of HSV-2–induced CXCL10 and CXCL11 in human cells, chemotaxis assay was performed using activated human peripheral blood mononuclear cells (PBMCs) and CD4^+^ T cells. The percentage of CD4^+^ cells was 95.6 and 33.4% in CD4^+^ T cells and PBMCs, respectively (Supplementary Material Figure 3). We also confirmed that CD4^+^ T cells and PBMCs were activated by PHA prior to the onset of chemotaxis assay (Supplementary Material Figure 4). ME180 cells were infected with HSV-2 for 24 h, and the chemotactic activity of supernatants was determined. In response to supernatants from HSV-2–infected cells, the migratory activity of PBMCs (Figure 3B) and CD4^+^ T cells (Figure 3C) was significantly increased. Cell migration was almost abolished upon the addition of anti-CXCL9 neutralizing Ab to supernatants from HSV-2–infected cells (Figures 3B,C), whereas a control Ab, anti-CXCL10 or CXCL11 neutralizing Ab did not have such effect, which is accordance with a previous study (26), indicating the critical role of CXCL9 in inducing CD4^+^ T cell migration. To further confirm the observation, a neutralizing Ab against CXCR3 was mixed well with cells for 1 h and then added into the upper chamber in chemotaxis assay, showing that the migration of CD4^+^ T cells was significantly reduced (Figure 3D). We also demonstrated that HSV-2 infection did not regulate the expression of CXCR3 by flow cytometry assay (Supplementary Material Figure 5A).The concentrations of CXCL9, CXCL10, and CXCL11 in the supernatants of HSV-2-infected ME180 cells used for chemotaxis assays were detected by CBA (Figure 3A), showing that CXCL10 and CXCL11 were produced at levels not less than that of CXCL9. We therefore conducted the chemotaxis assay using recombinant CXCL9, CXCL10, or CXCL11 at the similar concentration as that induced by HSV-2 infection. The results indicated that recombinant CXCL9 induced the migration of CD4^+^ T cells at a low concentration of 48 pg/ml, whereas CXCL10 and CXCL11 had no significant impact on CD4^+^ T cell migration at the concentrations of 55 pg/mL and 175 pg/mL, respectively (Figure 3E). Nevertheless, CXCL10 or CXCL11, at a much higher concentration than that induced by HSV-2 infection, did promote the migration of CD4^+^ T cells (Figures 3F,G), indicating that the concentrations of CXCL10 and CXCL11 required for CD4^+^ T cell migration are higher than that of CXCL9. Taken together, our results together indicated that HSV-2 infection-induced CXCL9 likely plays a predominant role in mediating CD4^+^ T cell migration.

### HSV-2 ICP4 Promotes the Production of Human CXCR3 Ligands

**Figure 4:**
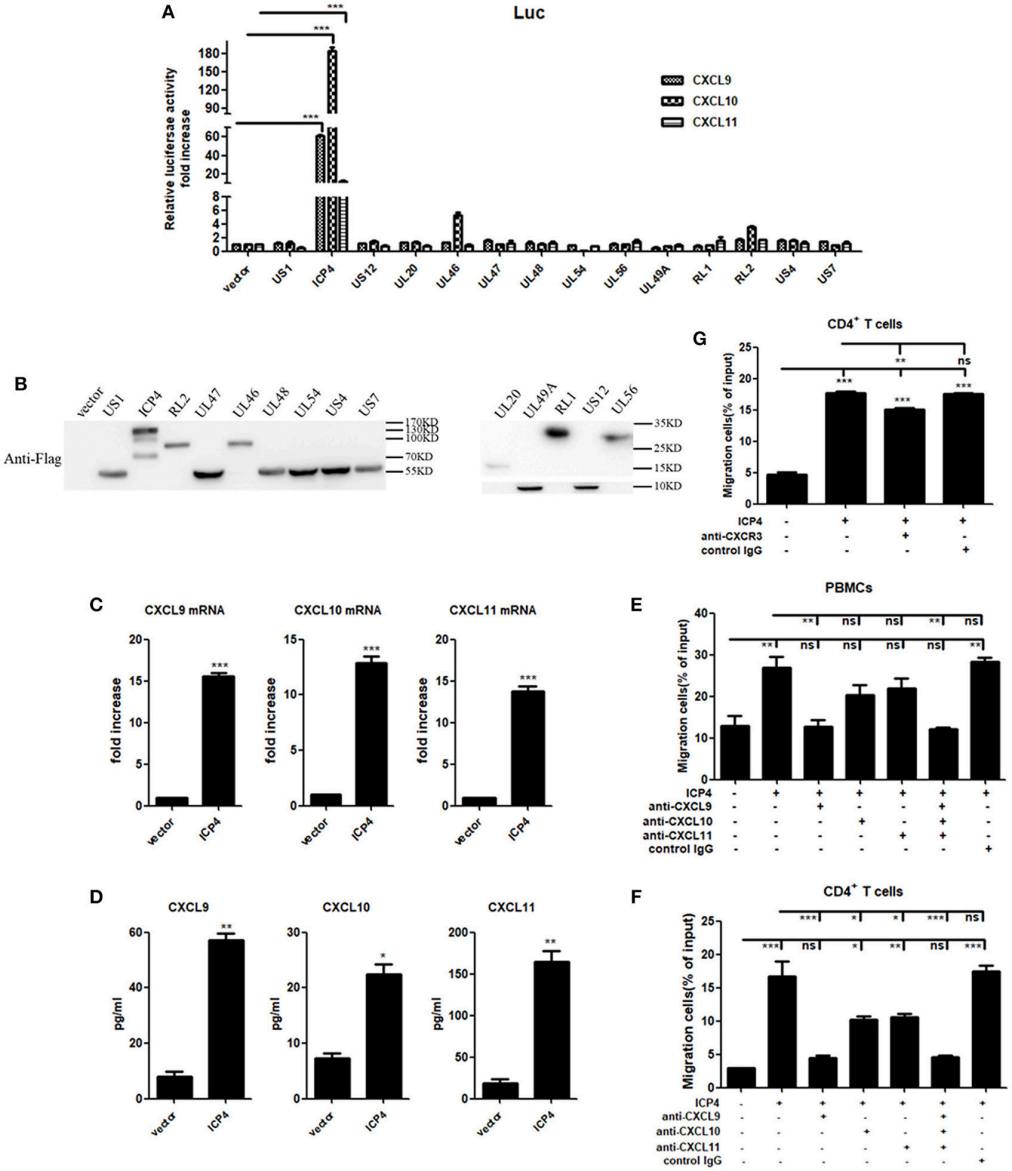
HSV-2 ICP4 promotes the production of human CXCR3 ligands. **(A)** ICP4 induces the activation of CXCR3 ligand promoters. ME180 cells in 24-well plates were transfected with 300 ng expression plasmid of HSV-2 gene or empty vector together with 150 ng CXCR3 ligand reporter and 15 ng phRL-TK. At 24 h post-transfection, DLR assay was performed. Values for the samples were normalized using Renilla luciferase values and expressed as fold increase of the value induced in cells transfected with empty vector. **(B)** The expression of HSV-2 genes was detected using anti-Flag Ab by Western Blot. ME180 cells were transfected with 3 μg HSV-2 gene expression plasmid for 24 h. The proteins were collected and detected using mouse anti-Flag Ab. **(C)** ICP4 induces the mRNA production of CXCR3 ligands. ME180 cells in 6-well plates were transfected with 3 μg ICP4 expression plasmid for 24 h. Cells were harvested and total RNA was extracted. The expression of CXCR3 ligands and GAPDH gene was evaluated by relative real-time quantitative PCR. The Ct values of GAPDH among all groups were equable and not overloaded. mRNA copies of CXCR3 ligands were normalized using GAPDH and expressed as fold increase of the value for the empty vector-transfected control. **(D)** ICP4 induces the production of CXCR3 ligands. As depicted in **(C)**, cell supernatants were collected, and the protein levels of CXCR3 ligands were measured by CBA. **(E,F)** CXCL9 induced by ICP4 recruits the migration of PBMCs **(E)** and CD4^+^ T cells **(F)**. ME180 cells in 6-well plates were transfected with 3 μg ICP4 expression plasmid for 24 h. Cell supernatants were collected and added to the lower chamber of transwell plates in the absence or presence of anti-CXCL9, –CXCL10, or/and –CXCL11 neutralizing Ab or control Ab for 1h. **(G)** Neutralization of CXCR3 reduces the migration of CD4^+^ T cells induced by ICP4. ME180 cells in 6-well plates were infected with HSV-2 at an MOI of 1 for 24 h. Cell supernatants were collected and added to the lower chamber of transwell plates. The activated CD4^+^ T cells were incubated with RPMI 1,640 medium containing anti-CXCR3 neutralizing Ab for 1 h and placed in the upper chamber. As depicted in Figure 3, cells migrated to lower chambers were counted. Cells migration was expressed as percentage of input. One representative out of three independent experiments is shown **(B)**. Data shown are mean ± S.D. of three independent experiments **(A,C–G)**. ns, not significant, *p < 0.05, **p < 0.01, ***p < 0.001.

UV-inactivation attenuated the ability of HSV-2 to induce CXCR3 ligand production, indicating that productive HSV-2 infection is necessary for the induced production of CXCR3 ligands. Given the complexity of HSV-2 genome which contains over 70 genes (27, 28), we next investigated which viral components are responsible for the induction of CXCR3 ligands during HSV-2 infection. ME180 cells were co-transfected with individual HSV-2 protein expression vector and the promoter reporter of CXCL9, CXCL10, or CXCL11. Dual luciferase reporter assay (DLR) indicated that HSV-2 immediate-early protein ICP4 significantly activated the promoters of CXCR3 ligands (Figure 4A). The expression of all tested viral proteins was assessed by Western Blot (Figure 4B). To further confirm the role of ICP4 on transcriptional activation, ME180 cells were transfected with ICP4 expressing plasmid, and cell supernatants were collected for CBA while mRNAs were extracted for reverse transcription PCR. Relative real-time quantitative PCR indicated that HSV-2 ICP4 enhanced the expression of CXCR3 ligands at mRNA level (Figure 4C). CBA showed that HSV-2 ICP4 enhanced the production of CXCR3 ligands at protein level (Figure 4D). Subsequent chemotaxis assay was performed to assess the role of ICP4-induced CXCR3 ligands in promoting the migration of activated PBMCs or CD4^+^ T cells. ME180 cells were transfected with ICP4 expressing plasmid, and the chemotactic activity of supernatants was determined. In response to supernatants from ICP4–transfected cells, the migratory activity of PBMCs (Figure 4E) and CD4^+^ T cells (Figure 4F) was significantly increased. Cell migration was significantly reduced upon the addition of anti-CXCL9 (Figures 4E,F) neutralizing Ab to supernatants from ICP4 expressing plasmid–transfected cells or anti-CXCR3 neutralizing Ab (Figure 4G) to cell suspension. In addition, the expression of CXCR3 in ICP4 expressing cells was also detected by flow cytometry assay, showing that ICP4 did not induce the expression of CXCR3 (Supplementary Material Figure 5B). These results indicated that the immediate-early protein ICP4 of HSV-2 promotes the production of human CXCR3 ligands, of which CXCL9 plays a predominant role in mediating CD4^+^ T cell migration.

### HSV-2 ICP4 Regulates the Expression of CXCR3 Ligands Via the p38 MAPK Signaling Pathway

**Figure 5:**
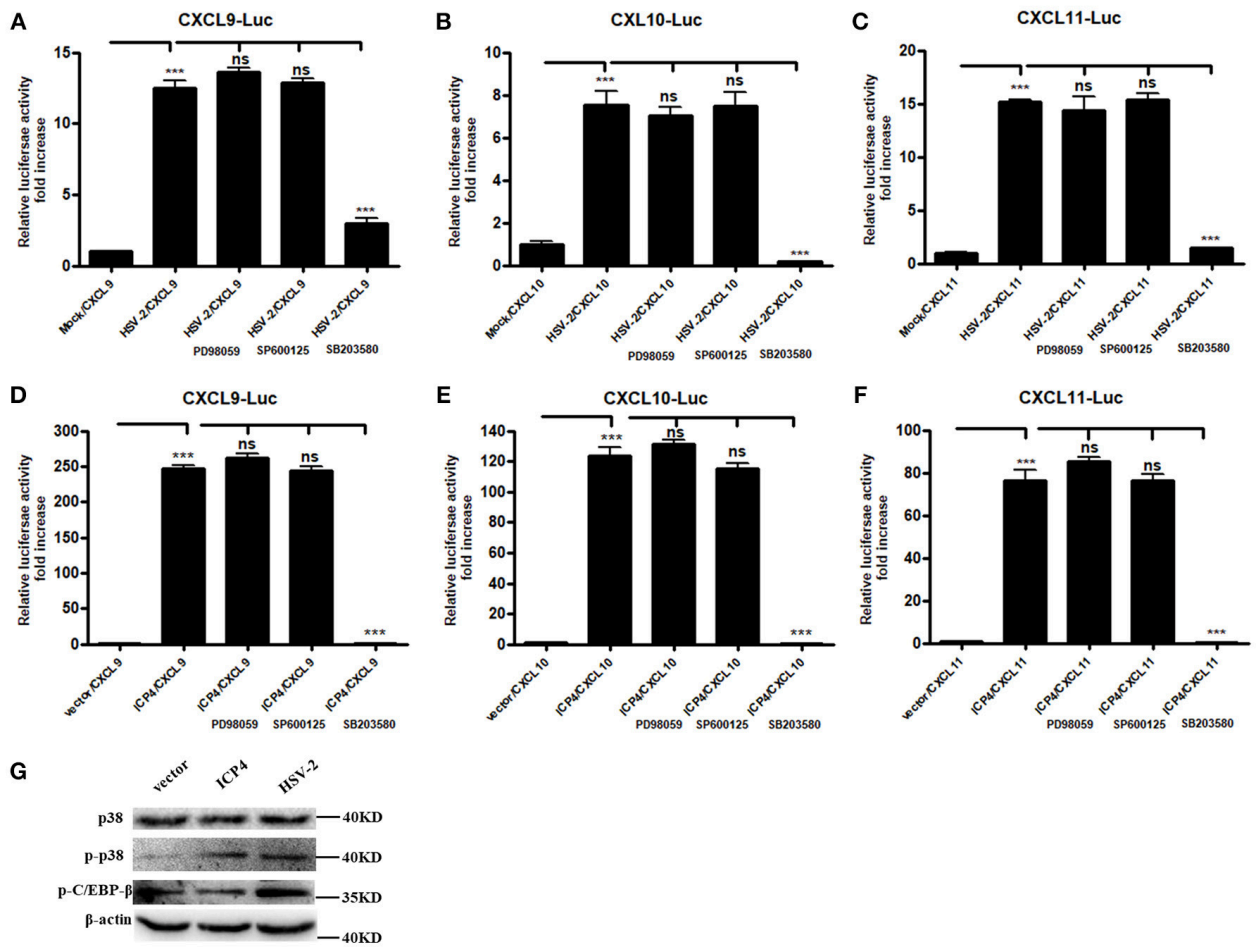
HSV-2 ICP4 regulates the expression of CXCR3 ligands via the p38 MAPK signaling pathway. **(A–C)** HSV-2 regulates the expression of CXCL9 **(A)**, CXCL10 **(B)**, and CXCL11 **(C)** via p38/MAPK signaling pathway. ME180 cells in 24-well plates were co-transfected with 150 ng CXCR3 ligand reporter and 15 ng phRL-TK. At 4 h post-transfection, cells were infected with HSV-2 at an MOI of 1 and supplemented with inhibitor PD98059, SP600125, or SB203580. DLR assay was performed at 24 h post-transfection. Values for the samples were normalized using Renilla luciferase values and expressed as fold increase of the value induced in mock-infected samples. **(D–F)** HSV-2 ICP4 regulates the expression of CXCL9 **(D)**, CXCL10 **(E)**, and CXCL11 **(F)** via p38/MAPK signaling pathway. ME180 cells in 24-well plates were co-transfected with 300 ng empty vector or ICP4 expression plasmid together with 150 ng CXCR3 ligand reporter and 15 ng phRL-TK. At 4 h post-transfection, cells were cultured in complete DMEM supplemented with inhibitor PD98059, SP600125, or SB203580. DLR assay was performed at 24 h post-transfection. Values for the samples were normalized using Renilla luciferase values and expressed as fold increase of the value induced in cells transfected with empty vector. **(G)** ICP4 activates p38 MAPK signaling pathway. ME180 cells were transfected with 3 μg ICP4 expression plasmid. The protein level of p38, phospho-p38 (p-p38) or phospho-C/EBP-β (p-C/EBP-β) was detected by Western Blot. Data shown are mean ± S.D. of three independent experiments **(A–F)**. ns, not significant, ***p < 0.001. One representative out of three independent experiments is shown **(G)**.

It is known that HSV-2 could activate MAPK pathway to regulate the expression of downstream genes (29). Our previous study demonstrated that HSV-2-mediated up-regulation of CXCL9 involves the p38 MAPK signaling pathway (14). To investigate whether MAPK pathway is involved in HSV-2-mediated transcriptional activation of CXCL10 and CXCL11 or ICP4-mediated transcriptional activation of CXCL9, CXCL10, and CXCL11, ME180 cells were pretreated with or without PD98059 (ERK inhibitor), SP600125 (JNK inhibitor) or SB203580 (p38 inhibitor), and then transfected with CXCR3 ligand reporter plasmid followed by infection with HSV-2 or co-transfected with CXCR3 ligand reporter plasmid and ICP4 expression plasmid. DLR assay showed that pretreatment of cells with SB203580, but not with PD98059 or SP600125, significantly decreased HSV-2–mediated activation of CXCL9 (Figure 5A), CXCL10 (Figure 5B), and CXCL11 (Figure 5C) promoters. In accordance, ICP4–mediated activation of CXCL9 (Figure 5D), CXCL10 (Figure 5E), and CXCL11 (Figure 5F) promoters was also decreased after pretreatment with SB203580. In our previous study, we found that HSV-2 infection could induce the phosphorylation of p38 and CCAAT/enhancer-binding protein-β (C/EBP-β). We then determined the impact of ICP4 on the activation of p38 MAPK pathway. ME180 cells were transfected with ICP4 expressing plasmid, and then the total or phosphorylation level of p38 and the phosphorylation level of C/EBP-β were examined by western blot assay. The results showed that, like HSV-2, ICP4 increased the phosphorylation level of p38 (Figure 5G). Taken together, these results suggest that HSV-2 ICP4–induced CXCR3 ligand expression in human cervical epithelial cells is mediated through the activation of p38 MAPK pathway.

### HSV-2 ICP4 Binds to the Promoters of CXCR3 Ligands by Interaction With TBP

**Figure 6:**
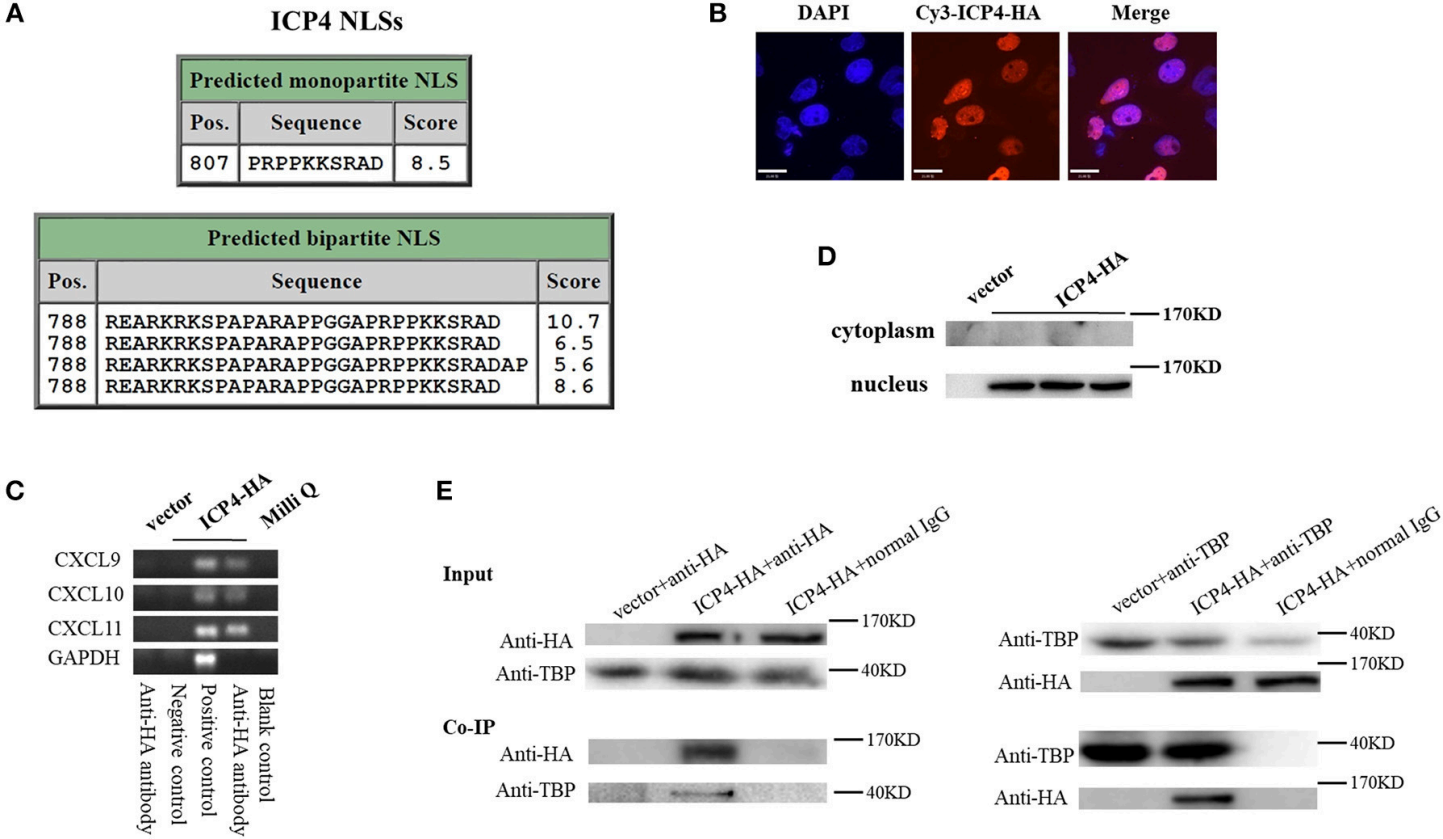
HSV-2 ICP4 binds to the promoters of CXCR3 ligands by interaction with TBP. **(A)** Schematic representation of the predicted NLSs of ICP4 amino acid (AA) sequence. **(B)** ICP4 is located in the nucleus. ME180 cells in 35-mm dishes with glass bottom were transfected with 2 μg empty vector or HA-tagged ICP4 expression plasmid for 24 h. Cells were stained with mouse anti-HA mAb, followed by Cy3-conjugated goat anti-mouse (red) as the secondary Ab. Cell nuclei (blue) were stained with DAPI. The images were obtained by fluorescence microscopy using 60 × objective. The scale bar indicates 21 μm. **(C)** The expression of ICP4 was stained using anti-HA mAb. **(D)** ICP4 binds to the promoters of CXCR3 ligands. ME180 cells were transfected with 3 μg empty vector or HA-tagged ICP4 expression plasmid for 24 h. Cells were lysed and subjected to ChIP assay using mouse anti-HA mAb, mouse anti-RNA polymerase II mAb (positive control) or mouse normal IgG (negative control) for immunoprecipitation. **(E)** ICP4 interacts with TBP. ME180 cells in 6-well plates were transfected with 3 μg empty vector or HA-tagged ICP4 expression plasmid for 24 h. Cells were lysed and subjected to co-immunoprecipitation (IP) using rabbit anti-HA or anti-TBP Ab. Rabbit normal IgG was used as a negative control. IP products and 5% input samples were examined using rabbit anti-HA and rabbit anti-TBP Abs by western blot. One representative out of three independent experiments is shown **(B–E)**.

Although HSV-2 ICP4 induces the expression of CXCR3 ligands via the p38 MAPK signaling pathway, it does not affect the phosphorylation level of C/EBP-β (Figure 5G), suggesting a novel mechanism involved in ICP4-mediated production of CXCR3 ligands. Previous studies have demonstrated that ICP4 is essential for virus growth (30) and functions as a transcriptional activator in some cases (31–34). It is probable that ICP4 binds to the promoters of CXCR3 ligands in the nucleus which results in their transcriptional activation. ICP4 must be located in the nucleus to act as a transcriptional factor. To test this hypothesis, we first analyzed the nucleotide sequence of ICP4, revealing several nuclear localization sequences (NLSs) (Figure 6A). ME180 cells were transfected with HA-tagged ICP4, and examined by indirect immunofluorescence (IF) and western blot assay, showing that ICP4 was indeed located in the nucleus (Figures 6B,C). In agreement, ICP4 was also located in the nucleus in the context of HSV-2 infection (Supplementary Material Figure 6). Meanwhile, at 24 h post-transfection with HA-tagged ICP4, ME180 cells were collected for chromatin immunoprecipitation (ChIP) assay. The ChIP assay indicated that ICP4 bound to the promoters of CXCR3 ligands (Figure 6D).

It is known that ICP4 can form a tripartite complex with transcription factor II B (TFIIB) and either TBP or transcription factor II D (TFIID) (35). TBP is required for the initiation of transcription by RNA polymerases I, II, and III, from promoters with or without a TATA box (36–38). TBP associates with a host of factors to form multi-subunit pre-initiation complexes on the core promoter. Through its association with different transcription factors, TBP can initiate transcription from different RNA polymerases (39). Considering that ICP4 induces the phosphorylation of p38 (Figure 5G), and that the transcriptional activation of TBP requires the activation of p38 MAPK signaling pathway (40, 41), ICP4-induced activation of p38 likely contributes to the transcriptional activation of TBP. To verify the interaction of ICP4 with TBP, ME180 cells were transfected with HA-tagged ICP4 for 24 h. Co-immunoprecipitation assays were performed to detect the interaction of ICP4 with TBP. The results indicated that ICP4 interacts with TBP as evidenced by using an anti-HA antibody to pulldown TBP or an anti-TBP antibody to pulldown ICP4 (Figure 6E). These data collectively indicated that HSV-2 ICP4 binds to the promoters of CXCR3 ligands by interaction with TBP, leading to the promoter activation of CXCR3 ligands.

## Discussion

Recruitment of CD4^+^ T cells, irrespective of their specificity, may significantly increase the chance of HIV-1 transmission (42, 43). Our previous study found that CXCL9 levels in cervical mucus from HSV-2–positive women were significantly increased and that HSV-2 infection induced CXCL9 expression in cervical epithelial cells (14). In addition, the expression of CXCL9 and CXCL10 was shown to be increased in the cervical tissues of mice infected by HSV-2 in studies to understand the contribution of recruited CD8^+^ T cells in control of HSV-2 infection (20), while CXCL9 induced by HSV-1 infection has been shown to recruit CD4^+^ T cells into the cornea (26). Moreover, circulating memory CD4^+^ T cells could migrate to the genital mucosa in mice challenged with HSV-2 (44). However, how HSV-2 infection affects the migration of CD4^+^ T cells at mucosal sites and the biological consequences remain to be fully determined.

In this study, we observed that, following vaginal challenge with HSV-2, mouse CXCR3 ligands CXCL9, CXCL10, and CXCL11 were all upregulated in the vaginal fluids of infected mice. In addition, CD4^+^ T cells migrated to the vaginal foci of infected mice, while the number of CD4^+^ T cells was significantly decreased after administration of CXCL9 neutralizing antibody to the vagina of mice. These indicate that HSV-2 infection can promote CD4^+^ T cell migration and this is mainly due to the induced CXCL9 expression. Although HSV-2 infection likely induces the expression of many other chemokines, our results showed that the migration of CD4^+^ T cells was significantly reduced after administration of a combination of neutralizing Abs against CXCL9, CXCL10, and CXCL11 into the vaginal tissue. In human cervical epithelial cells, we demonstrated that HSV-2 infection induced not only the production of CXCL9 but also that of CXCL10 and CXCL11. The common receptor for CXCL9, CXCL10, and CXCL11 is CXCR3, which can be rapidly induced on activated naive cells and sustain high level expression on Th1-type CD4^+^ T cells and effector CD8^+^ T cells (15). The other two ligands of CXCR3, CXCL4, and CXCL4L1, are released by platelets and have been implicated in atherogenesis and acute coronary syndrome (16). Therefore, we mainly focused on how HSV-2 infection enhances the production of CXCR3 ligands CXCL9, CXCL10, and CXCL11.

Early studies on transmitted/founder (T/F) HIV-1 have suggested that CD4^+^ T cells serve as the main target cells in the establishment of HIV-1 early infection (9, 45). Although CXCR3 ligands induced by HSV-2 can activate and recruit CD8^+^ T cells, these cells are specific for HSV-2 and may have an impact on the control of HSV-2 replication (20, 46, 47). In the current study, we mainly assessed the biological function of CXCR3 ligands induced by HSV-2 on CD4^+^ T recruitment. We found that chemokines induced by HSV-2 can mediate the migration of CD4^+^ T cells. Following experiments using neutralizing antibodies, the results indicated that the induced CXCL9 plays a crucial role in recruiting CD4^+^ T cells, which was further confirmed by using recombinant CXCL9, CXCL10, or CXCL11 at a concentration similar to that induced by HSV-2. Chemokines CXCL10 and CXCL11 at the concentrations around or higher than 300 pg/mL and 350 pg/mL, respectively, have been shown to have chemotactic activity for CXCR3^+^ cells (48–50), whereas CXCL9 has the same capability at a much lower concentration (14). We did not see significant reduction of CD4^+^ T cell migration when CXCL10 or CXCL11 was neutralized by the corresponding neutralizing antibody. One reasonable explanation is that the concentrations of CXCL10 and CXCL11 required for CD4^+^ T cell migration are much higher than that of CXCL9. In our study, the concentrations of CXCL10 and CXCL11 induced by ICP4 or HSV-2 were around or lower than 55 pg/mL and 175 pg/mL, respectively, which was unable to have an impact on CD4^+^ T cell migration as evidenced by the chemotaxis assay using recombinant CXCL10 and CXCL11. The recombinant CXCL10 at the concentration of 55 pg/mL had a marginal effect on CD4^+^ T cell migration, whereas the recombinant CXCL11 at the concentration of 175 pg/mL had no effect on the recruitment of CD4^+^ T cell. Compared to those induced by HSV-2 or ICP4, recombinant CXCL10 and CXCL11 at much higher concentrations chemotracted CD4^+^ T cells in a dose-dependent manner. Although beyond the scope of this current study, it will be important to address the roles of CXCR3 ligands in mediating CD4^+^ T cell migration and HIV-1 mucosal transmission when an animal model become available to study HSV-2 and HIV-1 co-infection.

We found that UV-inactivated HSV-2 did not significantly induce the transcriptional activation of CXCL9 and CXCL10. Although CXCL11 appeared to be induced by UV-inactivated HSV-2, the level of induction was low. These results indicate that productive infection of HSV-2 is essential for the induced production of CXCR3 ligands. HSV-2 genome contains over 70 genes (27, 28). Following screening a range of HSV-2 ORFs, we identified the immediate-early protein ICP4 as the key viral component in inducing the expression of CXCR3 ligands. ICP4 was barely detectable when cells were treated with UV-inactivated HSV-2, further suggesting the importance of ICP4 in inducing CXCR3 ligand expression. In agreement, we observed the co-localization of ICP4 with CXCL9, CXCL10 or CXCL11 in the mouse vaginal epithelial layer by immunofluorescence histochemistry assay (Supplementary Material Figure 8). ICP4-induced CXCL9 played a crucial role in the chemotaxis of CD4^+^ T cells, which is in accordance with that induced by HSV-2. We previously found that HSV-2 infection-induced CXCL9 expression is regulated by the transcriptional factor C/EBP-β (14). In the current study, we found that ICP4 did not affect the phosphorylation of C/EBP-β, although ICP4 induced the production of all the three CXCR3 ligands via p38 MAPK signaling pathway. These together indicate a novel mechanism underlying ICP4-induced CXCR3 ligand production, and that other viral component(s) is likely to be involved in the phosphorylation of C/EBP-β. It is known that ICP4 is a major transcriptional activator and essential for progression beyond the immediate-early phase of infection (28). Indeed, we successfully constructed a ICP4-null HSV-2 bacterial artificial chromosome (BAC) but were unable to rescue the ICP4-null HSV-2 (data not shown), further strengthening the essential role of ICP4 in the regulation of viral gene expression. ICP4 can function as a transcriptional activator in some cases (31–34). It may act as a transcriptional activator to induce the activation of CXCR3 ligand promoter. To function as a transcriptional factor, ICP4 needs to be in the nucleus where it can bind to the promoters of CXCR3 ligands. We found that ICP4 is indeed located in the nucleus and can bind to the promoters of CXCR3 ligands, resulting in the expression of corresponding chemokines. ICP4 was also located in the nucleus in the context of HSV-2 infection. We also observed the interaction of ICP4 with TBP, which could contribute to the binding of ICP4 to the promoters of CXCR3 ligands. Nevertheless, ICP4 seems not to serve as a consensus transcription factor to activate gene expression, as ICP4 did not activate the promoters of other cytokines including TNF, IL-6 (Supplementary Material Figure 7).

In conclusion, we first found that the expression of CXCR3 ligands CXCL9, CXCL10, and CXCL11 was induced following mice vaginally challenged with HSV-2, which was associated with the increased number of CD4^+^ T cells in the vaginal foci of infected mice as well as CXCL9-mediated cell migration. We further observed that HSV-2 infection induced the production of CXCL10 and CXCL11 in addition to CXCL9 in human cervical epithelial cells. Although CXCL10 and CXCL11 could be induced by HSV-2, HSV-2-induced CXCL9 played a critical role in recruitment of CD4^+^ T cells. Mechanistically, after identifying HSV-2 ICP4 as a vital viral component in inducing CXCR3 ligands, we demonstrated the contribution of ICP4-induced CXCL9 in recruiting CD4^+^ T cells and a critical role played by p38 MAPK signaling pathway in HSV-2 infection- or ICP4–induced CXCR3 ligand expression. HSV-2 ICP4 binds to the corresponding promoters of CXCR3 ligands by interaction with TBP to activate their transcription. Our study together reveals the molecular mechanism underlying HSV-2-induced CD4^+^ T cell accumulation in mucosal infection sites, which may be crucial for understanding HSV-2 infection-enhanced HIV-1 sexual transmission and the development of intervention strategies.

## Author Contributions

MZ and QH conceived the study. MZ, XD, XG, BZ, RC, DZ, and MF conducted experiments. MZ conducted experiments in Figures 1–6. XG extracted the cytoplasmic and nuclear proteins in Figure 5. BZ and RC conducted western blot experiment in Figures 5, 6, respectively. XD, DZ, and MF provided help in conducting mouse experiments in Figure 1. LG, KH, ML, and YL offered advices and technical assistance. HH provided help in the construction of Flag-tagged plasmids. MZ, SG, and QH analyzed the data. MZ and QH wrote the manuscript. All authors reviewed the manuscript.

### Conflict of Interest Statement

The authors declare that the research was conducted in the absence of any commercial or financial relationships that could be construed as a potential conflict of interest.

## Abbreviations

HSV-2: herpes simplex virus type 2
HIV-1: human immunodeficiency virus type 1
AIDS: acquired immune deficiency syndrome
PBMC: peripheral blood monocyte cell
C/EBP-β: CCAAT/enhancer-binding protein-β
MIG: monokine induced by gamma-interferon
IP-10: interferon-induced protein-10
I-TAC: interferon-inducible T-cell alpha chemoattractant
TFIIB: transcription factor II B
TBP: TATA binding protein
TFIID: transcription factor II D
Ultraviolet: UV.
