# Supplementary material for: Herpes Simplex Virus Type 2 Infection-Induced Expression of CXCR3 Ligands Promotes CD4+ T Cell Migration and Is Regulated by the Viral Immediate-Early Protein ICP4
Source: Front Immunol. 2018 Dec 19;9:2932. doi: 10.3389/fimmu.2018.02932 (PMC6305738; doi:10.3389/fimmu.2018.02932)
Supplement: Supplementary file 1 [file Data_Sheet_1.docx]

Supplementary Material

Herpes Simplex Virus-2 infection-induced expression of CXCR3 ligands promotes CD4^+^ T cell migration and is regulated by the viral immediate-early protein ICP4

Mudan Zhang^1^, Xu Deng^2, 3^, Xinmeng Guan^2, 3^, Lanlan Geng^4^, Ming Fu^2, 3^, Binman Zhang^2, 3^, Rui Chen^2^, Huimin Hu^2, 3^, Kai Hu^2^, Di Zhang^2, 3^, Mei Li^2, 3^, Yalan Liu^2^, Sitang Gong^4 *^, Qinxue Hu^2, 5 *^

^1^ The Joint Center of Translational Precision Medicine: 1. Guangzhou Institute of Pediatrics, Guangzhou Women and Children’s Medical Center, Guangzhou, China; 2. Wuhan Institute of Virology, Chinese Academy of Science, Wuhan, China

^2^ State Key Laboratory of Virology, Wuhan Institute of Virology, Chinese Academy of Sciences, Wuhan, China

^3^ University of Chinese Academy of Sciences, Beijing, China

^4^ Department of Gastroenterology, Guangzhou Women and Children's Medical Center, Guangzhou Medical University, Guangzhou, China.

^5^ Institute for Infection and Immunity, St George’s University of London, London, UK

*** Correspondence:** Dr. Qinxue Hu: [qhu@wh.iov.cn](mailto:qhu@wh.iov.cn) Dr. Sitang Gong: [sitangg@126.com](mailto:sitangg@126.com)

# Supplementary Figures and Tables

## Supplementary Tables

| **Table S1. Primers used in this study** | | | | |  |
| --- | --- | --- | --- | --- | --- |
|  |  |  |  |  |  |
| For plasmid construction | | | | |  |
| Primer Name | | Sequence^a^ (5'-3') | Cloning Sites | |  |
| UL20-F | CA**GAATTC**ATGACAATGCGGGATGATG | | | EcoR I | |
| UL20-R | AT**CTCGAG**TTAGAACGCGACGGGTGCCT | | | Xho I | |
| RS1-HA-F | CG**GCTAGC**CACCATGTACCCATACGATGTTCCAGATTACGCTATGTCGGCGGAGCAGCGGAA | | | Nhe I | |
| RS1-Flag-F | CGGCTAGCATGGATTACAAGGATGACGACGATAAGTCGGCGGAGCAGCGGAA | | | Nhe I | |
| RS1-HA-R | CC**AAGCTT**TCACTCCCCAAACAGTCCGTCGTCGTCGTCCTCCAGCTCCGCGTCCATG | | | Hind III | |
| UL46-F | GC**AAGCTT**CGAACGGTCTCCGCCATGCAACG | | | Hind III | |
| UL46-R | TA**TCTAGA**CGGTCGCATTTTTTTAATGGCTCTG | | | Xba I | |
| UL47-F | CC**AAGCTT**ATGTCCGTGCGCGGGCAT | | | Hind III | |
| UL47-R | AA**TCTAGA**GGATATAGGGTTGCTTTTTA | | | Xba I | |
| UL48-F | CC**AAGCTT**ATGGACCTGTTGGTCGACG | | | Hind III | |
| UL48-R | AA**TCTAGA**CTACCCCCCAAAGTCGTCAATG | | | Xba I | |
| UL56-F | CA**GAATTC**ATGGCGTTGGGGGCTGGGCA | | | EcoR I | |
| UL56-R | AC**CTCGAG**TTACCGCCAAAGGAAGGCCA | | | Xho I | |
| UL49A-F | CC**AAGCTT**ATGACGGGGAAACCCGCAAG | | | Hind III | |
| UL49A-R | AA**GAATTC**TCAGGCTGACCCGGCGGCCA | | | EcoR I | |
| US4-F | CA**GGATCC**ATGCACGCCATCGCTCCCAG | | | Bam HI | |
| US4-R | TT**CTCGAG**CTAATCCCGCTCGGGTGGCA | | | Xho I | |
| US7-F | AC**GATATC**CCGTCAGCTAGTCTCCGATC | | | EcoR V | |
| US7-R | CA**TCTAGA**GTCGGAAGACGCCTTACCAG | | | Xba I | |
| RL1-F | CC**AAGCTT**GCCGAGCCCAGCCGCCCGCCAT | | | Hind III | |
| RL1-R | AA**TCTAGA**GCCGCCCTCGCCGGTTCAA | | | Xba I | |
| UL20-Flag-F | CAGAATTCATGGATTACAAGGATGACGACGATAAGACAATGCGGGATGATG | | | Hind III | |
| UL20-Flag-R | ATCTCGAGTTAGAACGCGACGGGTGCCTTCAAGATGGCCCTGGTCCAAAA | | | Xba I | |
| UL46-Flag-F | GCAAGCTTCGAACGGTCTCCGCCATGGATTACAAGGATGACGACGATAAGCAACG | | | Hind III | |
| UL46-Flag-R | TATCTAGACGGTCGCATTTTTTTAATGGCTCTGGTGTCGGCCGCGTTTGAGCT | | | Xba I | |
| UL47-Flag-F | CCAAGCTTATGGATTACAAGGATGACGACGATAAGTCCGTGCGCGGGCAT | | | Hind III | |
| UL47-Flag-R | AATCTAGAGCTATGGGCGTGGCGGGCCGCCCAGCCCGGTCGCG | | | Xba I | |
| UL48-Flag-F | CCAAGCTTATGGATTACAAGGATGACGACGATAAGGACCTGTTGGTCGACG | | | EcoR I | |
| UL48-Flag-R | TCTAGACTACCCCCCAAAGTCGTCAATGCCCATGGCATCGGTAAACATCT | | | Xho I | |
| UL49A-Flag-F | CCAAGCTTATGGATTACAAGGATGACGACGATAAGACGGGGAAACCCGCAAG | | | Hind III | |
| UL49A -Flag-R | AAGAATTCTCAGGCTGACCCGGCGGCCAGTGCCCGCTGACATATCTGATACA | | | EcoR I | |
| UL56-Flag-F | CAGAATTCATGGATTACAAGGATGACGACGATAAGGCGTTGGGGGCTGGGCA | | | Bam HI | |
| UL56-Flag-R | ACCTCGAGTTACCGCCAAAGGAAGGCCAAGATGATAACGACGACCA | | | Xho I | |
| US4-Flag-F | CAGGATCCATGGATTACAAGGATGACGACGATAAGCACGCCATCGCTCCCAGGTT | | | EcoR V | |
| US4-Flag-R | TTCTCGAGCTAATCCCGCTCGGGTGGCAGACATACGTAACGCACGCTCGGGT | | | Xba I | |
| US7-Flag-F | ACGATATCATGGATTACAAGGATGACGACGATAAGGCTCGCGGGGCCGGGTT | | | Hind III | |
| US7-Flag-R | CATCTAGAGTCGGAAGACGCCTTACCAGAGGACGGACGGATAGGAGGCC | | | Xba I | |
| RL1-Flag-F | CCAAGCTTATGGATTACAAGGATGACGACGATAAGTCCCGCCGCCGGGGTCCCC | | | Hind III | |
| RL1-Flag-R | AATCTAGACTAGACCGCCCGACGGCCCGGGCCCGCGGCGGCGGAGGACC | | | Xba I | |
| CXCL10 Luc-F | 5’-CG**GAGCTC**ACTATGAATAATCAGTCAAG-3’ | | | Sac I | |
| CXCL10 Luc-R | 5’-AA**AAGCTT**GGTGCTGAGACTGGAGGTTC-3’ | | | Hind III | |
| CXCL11 Luc-F | 5’-AA**GAGCTC**ATCACATGAGCAAAGAGGA-3’ | | | Sac I | |
| CXCL11 Luc-R | 5’-CG**CTCGAG**GTTTGTTTTTTGCTGTTGC-3’ | | | Xho I | |
| For real time PCR | | | | |  |
| CXCL9-F | 5'-TCCTCTTGGGCATCATCTTGCTGGTT-3' | |  | |  |
| CXCL9-R | 5'-GGATTGTAGGTGGATAGTCCCTTGGTTG-3' | |  | |  |
| CXCL10-F | 5'-CATTCTGATTTGCTGCCTTATCTTTC-3' | |  | |  |
| CXCL10-R | 5'-GACCTTTCCTTGCTAACTGCTTTCA-3' | |  | |  |
| CXCL11-F | 5'-AAGCAGTGAAAGTGGCAGATATTGAGA-3' | |  | |  |
| CXCL11-R | 5'-TTGGGATTTAGGCATCGTTGTCC-3' | |  | |  |
| GAPDH-F | 5'-GGGAAGCTCACTGGCATGG-3' | |  | |  |
| GAPDH-R | 5'-TTACTCCTTGGAGGCCATGT-3' | |  | |  |
| For ChIP assay | | | | |  |
| CXCL9 pro-F | 5'-CAAGTTTGTGGTCAATTTAG-3' | |  | |  |
| CXCL9 pro-R | 5'-CTTTAGAGAACACATTTTGG-3' | |  | |  |
| CXCL10 pro-F | 5'-GGTGCTGAGACTGGAGGTTC-3' | |  | |  |
| CXCL10 pro-R | 5'-TTGCCAGTTCCAGATCTTTG-3' | |  | |  |
| CXCL11 pro-F | 5'-GTTTGTTTTTTGCTGTTGC-3' | |  | |  |
| CXCL11 pro-R | 5'-GAGGGAAATTCCTGTGCCAT-3' | |  | |  |
| GAPDH-F | 5'-GGGAAGCTCACTGGCATGG-3' | |  | |  |
| GAPDH-R | 5'-TTACTCCTTGGAGGCCATGT-3' | |  | |  |

## Supplementary Figures


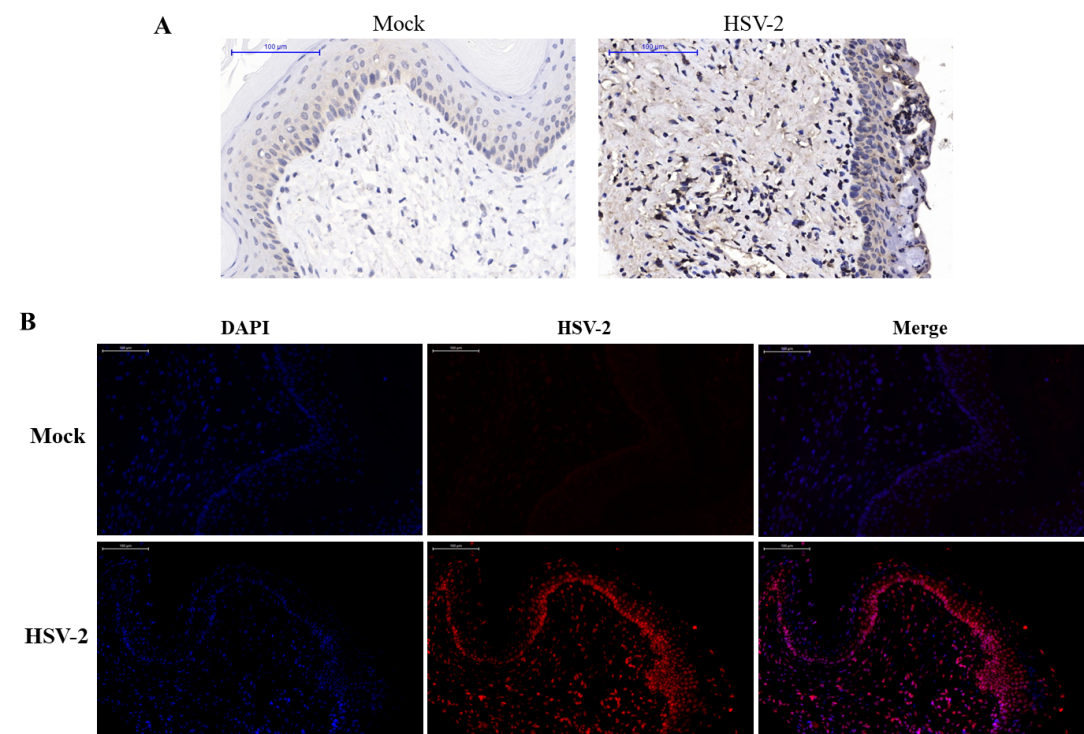


**Supplementary Figure 1.** Vaginal infection of HSV-2 in mice. Seven days prior to HSV-2 challenge, BALB/c mice were injected with progesterone in multiple sites. Mice were then anesthetized with pentobarbital sodium and challenged intravaginally with 10μL/mouse HSV-2 at a concentration of 6×10^7^ PFU/ml. Mice mock-infected with medium alone were set as background controls. Cervical-vaginal tissues were collected at day 7 after challenge. HSV-2 productive infection in mouse vagina was detected using anti-HSV-2 pAb by IHC (A) and immunofluorescence histochemistry assay (B). One representative experiment out of three is shown. The scale bar indicates 100 μm. One representative out of three independent experiments is shown.


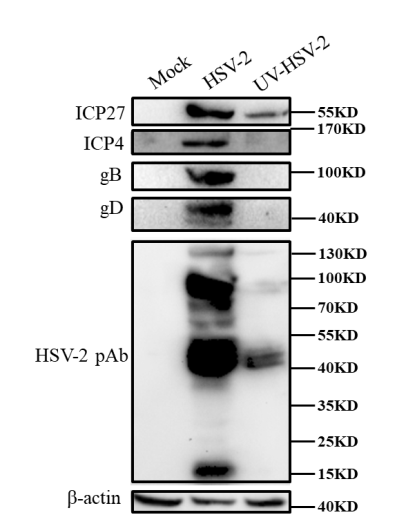


**Supplementary Figure 2.** Detection of viral protein expression following HSV-2 infection. ME180 cells in 6-well plates were infected with HSV-2 or ultraviolet-inactivated HSV-2 (UV-HSV-2) at an MOI of 1 for 24 h. Cells mock-infected with medium alone were set as the control. The protein levels of ICP27, ICP4, gB, and gD of HSV-2 were detected by Western Blot. One representative out of three independent experiments is shown. pAb, polyclonal antibody.


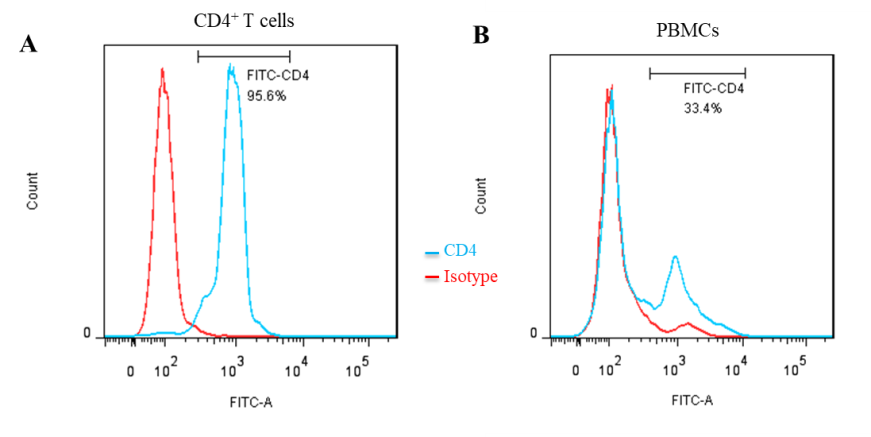


**Supplementary Figure 3.** Percentage of CD4^+^ cells in CD4^+^ T cells and PBMCs. CD4^+^ T cells (A) and PBMCs (B) were incubated with BB515 conjugated anti-human CD4 Ab followed by flow cytometry. Background staining was assessed by isotype-matched control Ab. One representative experiment from one donor out of three is shown.


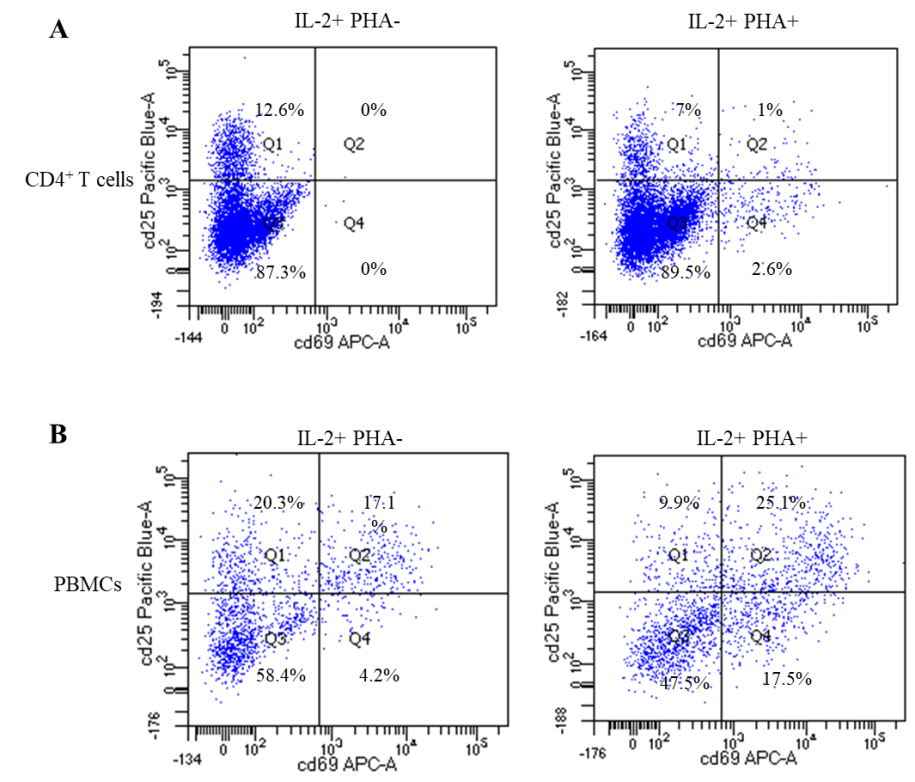


**Supplementary Figure 4.** Activation of CD4^+^ T cells and PBMCs by PHA. CD4^+^ T cells (A) and PBMCs (B) were incubated with BB515 conjugated mouse anti-human CD4 Ab, BV421 conjugated mouse anti-human CD25 Ab and APC conjugated mouse anti-human CD69 Ab followed by flow cytometry. Background staining was assessed by isotype-matched control Abs. BB515 positive CD4^+^ cells were first gated, and then the expression of CD25 and CD69 was analyzed. One representative experiment from one donor out of three is shown.


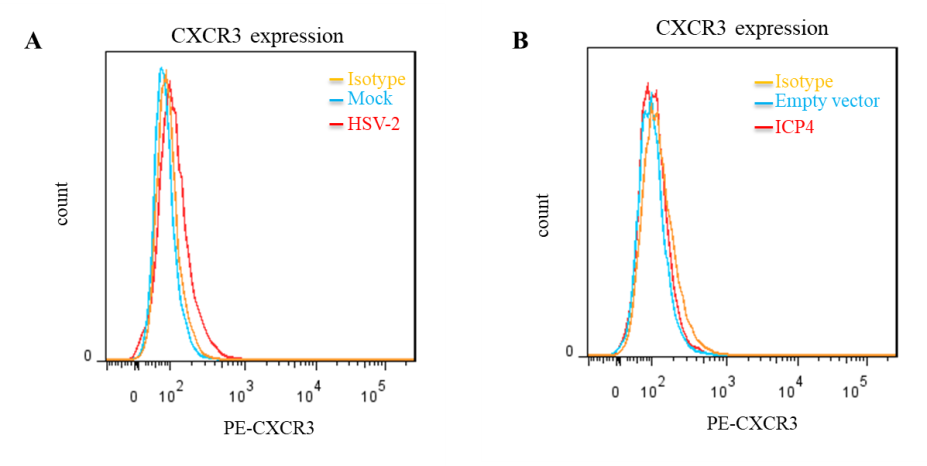


**Supplementary Figure 5.** The expression of CXCR3 is not induced by HSV-2 (A) or ICP4 (B). PM1 cells were infected with HSV-2 at an MOI of 1, or electro-transfected with 3 μg ICP4 expression plasmid or empty vector for 24 h. Cells mock-infected with medium alone were set as the control. The expression of CXCR3 was detected using PE conjugated anti-human CXCR3 Ab by flow cytometry. Background staining was assessed by isotype-matched control Ab. One representative experiment from one donor out of three is shown.


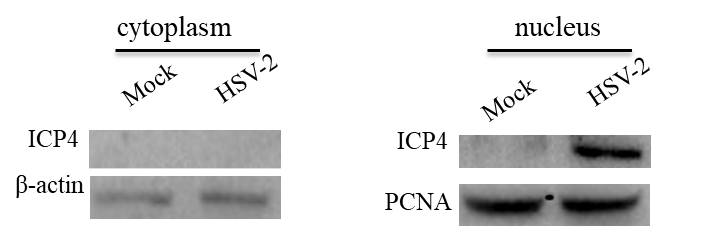


**Supplementary Figure 6.** ICP4 is located in the nucleus in the context of HSV-2 infection. ME180 cells were infected with HSV-2 at an MOI of 1 for 24 h. Cells mock-infected with medium alone were set as the control. The cytoplasmic and nuclear proteins were isolated. The presence of ICP4 was stained using anti-ICP4 Ab followed by Western Blot. One representative out of three independent experiments is shown.


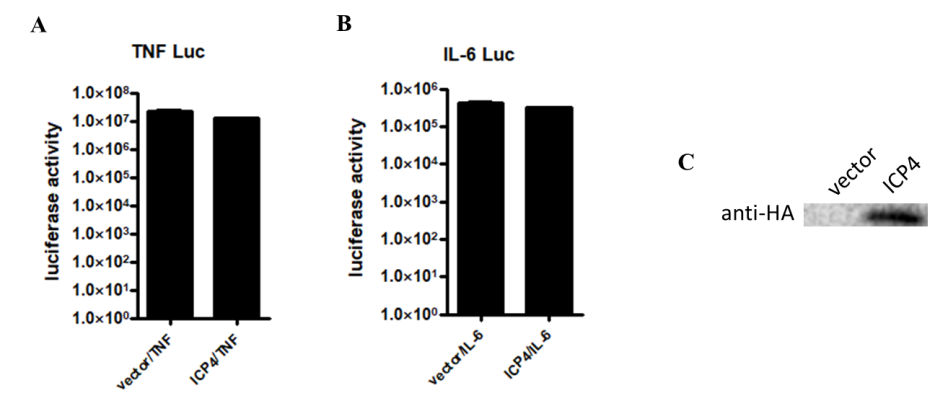


**Supplementary Figure 7.** ICP4 does not activate the promoter of TNF (A) or IL-6 (B). ME180 cells in 24-well plates were transfected with 300 ng ICP4 expression plasmid or empty vector together with 150 ng TNF-Luc or IL-6 Luc and 15 ng phRL-TK for 24 h. Luciferase reporter assay was performed. The expression of ICP4 was confirmed by western blot (C). Data shown are mean ± S.D. of three independent experiments (A, and B). One representative out of three independent experiments is shown (C).

**
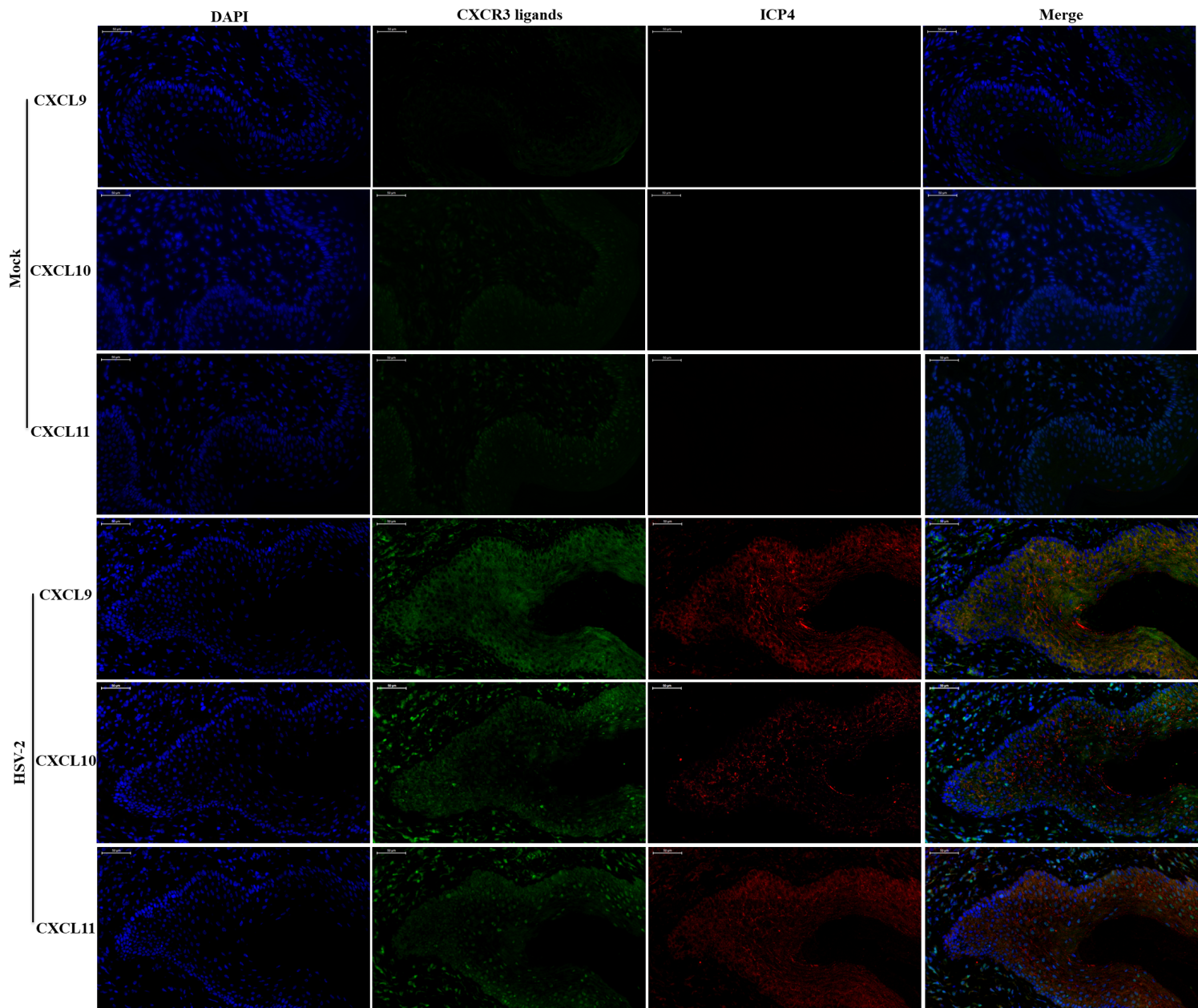
**

**Supplementary Figure 8.** CXCR3 ligands co-localize with ICP4 in the mouse vaginal epithelial layer. Seven days prior to HSV-2 challenge, BALB/c mice were injected with progesterone in multiple sites. Mice were then anesthetized with pentobarbital sodium and challenged intravaginally with 10 μL/mouse HSV-2 at a concentration of 6×10^7^ PFU/ml. Mice mock-infected with medium alone were set as background controls. Cervical-vaginal tissues were collected at day 7 after challenge. CXCR3 ligands and HSV-2 ICP4 were stained by anti-CXCL9, -CXCL10 or -CXCL11 Ab together with anti-ICP4 Ab by immunofluorescence histochemistry experiments. One representative out of three independent experiments is shown. The scale bar indicates 50μm.
